# Analysis of PM-bound polycyclic aromatic hydrocarbons exposure among motorcycle taxi drivers in six central provinces in Thailand in winter

**DOI:** 10.1371/journal.pone.0336587

**Published:** 2025-12-01

**Authors:** Kamonwan Samana, Kimihito Ito, Orasa Suthienkul, Arroon Ketsakorn

**Affiliations:** 1 Faculty of Public Health, Thammasat University, Pathum Thani, Thailand; 2 Division of Bioinformatics, International Institute for Zoonosis Control, Hokkaido University, Sapporo, Japan; 3 Faculty of Public Health, Mahidol University, Bangkok, Thailand; Nanjing University of Information Science and Technology, CHINA

## Abstract

Motorcycle taxis are popular transportation in areas with heavy traffic in Thailand. In this study, we recruited motorcycle taxi drivers in six central provinces in Thailand between January and March 2023 and measured their particulate matter (PM) and PM-bound polycyclic aromatic hydrocarbon (PAH) exposures using personal air sampling. We found that the PM_10_ and PM_2.5_ concentrations measured by personal air sampling were independent of those monitored at air quality monitoring stations or by area air sampling devices. Among the six provinces, motorcycle taxi drivers in Pathum Thani were exposed to the highest mean concentration of PM_10_ (224.9 µg/m^3^), PM_2.5_ (410.9 µg/m^3^), PM_10−_bound total PAH (38.4 ng/m^3^), and PM_2.5_-bound total PAH (36.9 ng/m^3^). Four workstations (PTT-1 to PTT-4) using 22 samples of PM₁₀ and 25 samples of PM₂.₅ personal air samplers showed unexpectedly higher PM₂.₅ than PM₁₀, likely due to route-specific environmental factors, as drivers follow variable routes determined by passenger destinations and daily demand. The incremental lifetime cancer risk of PM_10−_bound PAH and PM_2.5_-bound PAH in Pathum Thani were 4.5 × 10^−8^ and 7.8 × 10^−8^, respectively, which were acceptable levels. None of the individuals’ lung function parameters was significantly correlated with the individuals’ concentrations of PM_10_, PM_2.5_, PM_10−_bound total PAH, or PM_2.5_-bound total PAH. However, province averages of PM_10_-bound total PAH exposure of motorcycle taxi drivers were positively correlated with the proportions of participants who answered symptoms of chronic bronchitis in the province. The causal relationship between motorcycle taxi drivers’ PM and PM-bound PAH exposure in Pathum Thani and their respiratory symptoms needs to be further investigated.

## Introduction

People working in areas with heavy traffic have a potential risk of being exposed to diesel exhaust from motor vehicles. The major harmful chemicals from diesel exhaust are particulate matter (PM) and polycyclic aromatic hydrocarbons (PAHs). Several cities with heavy traffic were reported to have a high concentration of particulate matter with an aerodynamic diameter of less than or equal to 10 µm (PM_10_), particulate matter with an aerodynamic diameter of less than or equal to 2.5 µm (PM_2.5_) [1–3], and PAH [4–6].

Thailand had an annual mean concentration of PM_2.5_ of 26.3 µg/m^3^, and it was ranked 27^th^ highest among 200 cities worldwide in 2019 [7]. Previous studies reported that vehicle emissions were the major PM source in urban areas in Thailand [8–10]. Traffic emissions from four-stroke motorcycles, tuk-tuks, compressed natural gas vehicles, and two-stroke motorcycles were the source of PAHs in Bangkok [11].

Previous studies reported that the incremental lifetime cancer risk (ILCR) from PM-bound PAH concentration in some urban areas were potential risk levels (10^−6^ − 10^−4^). The ILCR of PM_2.5_-bound PAHs in urban residential areas in China was 7.45 × 10^−5^ [12]. The ILCR of PM_2.5_-bound PAHs in central Tehran was 2.8 × 10^−5^ [13]. The ILCR of PM_2.5_-bound PAHs was 3.03 × 10^−4,^ with vehicle emissions contributing 57.1% (1.6 × 10^−4^) to the total risk on average in China [14]. The ILCR of PM_2.5_-bound PAHs in Iran was 1.33–2.28 × 10^−5^, and heavy-duty vehicles and natural gas-coal/biomass combustion emissions area had the highest cancer risk than the other apportioned sources [15]. The ILCR of PM_2.5_-bound PAHs in adults within area air sampling of automobile workshops in Benin City, Nigeria, were 3.58 × 10^−5^ and 2.80 × 10^−5^ for dry and wet seasons, respectively [16]. These studies measured the PM and PM-bound PAH concentrations by ambient air sampling at monitoring stations.

Some studies have investigated PM exposure by personal air sampling. Personal air sampling can measure an individual’s exposure, which may depend on the individual’s activity. Commuting in winter in Greece by bicycle (99.8 µg/m^3^) was the highest exposure to PM_4,_ followed by bus (91.0 µg/m^3^) and car (37.2 µg/m^3^) [17]. Travel by scooters in Taiwan had the highest exposure to PM_2.5_ (53.6 µg/m^3^), followed by walking (42.7 µg/m^3^), bus (34.7 µg/m^3^), car (26.2 µg/m^3^), and subway (21.7 µg/m^3^) [18]. Walking on the street level in Kenya had higher PM_2.5_ exposure (119.5 µg/m^3^) than walking on the third-floor rooftop (42.8 µg/m^3^) [19]. Commuting motorcyclists in Taipei (27.65 µg/m^3^) had higher exposure to PM_2.5_ than cyclists (23.27 µg/m^3^) [20].

The effects of PM exposure on the health of outdoor workers have been investigated. PM_2.5_ exposure among policemen in Malaysia was significantly associated with lung function parameters [21]. The lung function parameters of traffic police officers in India decreased significantly compared to a control group [22]. In contrast, no decline in lung function was associated with exposure to PM_10_ among subway workers in France [23]. A comprehensive summary of prior studies examining PM exposure and incremental lifetime cancer risk (ILCR) is provided in S1 Table.

Motorcycle taxis are popular transportation in areas with heavy traffic in Thailand. A total of 135,231 motorcycle taxi drivers were estimated to work in Bangkok and adjacent provinces [24]. Motorcycle taxi drivers can represent outdoor workers with a high risk of PM and PAH exposure in areas with heavy traffic in Bangkok and adjacent provinces. However, PM-bound PAH exposure among motorcycle taxi drivers in Thailand has not been studied previously. Motorcycle taxi drivers’ exposure to PM-bound PAH needs to be investigated to assess the impact of PM-bound PAH on their health.

In our previous study, we recruited motorcycle taxi drivers in six central provinces in Thailand and conducted personal air sampling in the rainy season, and we investigated the relationship between motorcycle taxi drivers’ respirable dust exposure and their lung function parameters [25]. In another paper, we assessed health risks from exposure to respirable dust using the same data [26]. Personal air sampling in these previous studies measured only PM_4_ concentration, and the PM-bound PAH was not measured. In this study, we measured the concentration of PM_2.5_ and PM_10_ as well as PM-bound PAH concentration by personal air sampling in the same provinces in Thailand in the winter season to estimate the incremental lifetime cancer risk for motorcycle taxi drivers. This is the first study that measured PM-bound total PAHs concentration using personal air sampling in Thailand. Furthermore, we analyze the association between PM-bound PAH exposure among motorcycle taxi drivers and their health status, such as symptoms of respiratory diseases and lung function parameters.

## Materials and methods

### Study area

This study was conducted in six central provinces in Thailand: Bangkok, Nonthaburi, Pathum Thani, Samut Prakan, Samut Sakhon, and Nakhon Pathom. The population of these six provinces accounts for 10,872,100 persons, with a density of 1400.74 per square kilometre, which is approximately 10.9 times higher than the average density of Thailand (128.95 population per square kilometre). A total of 12,535,879 automobiles, including cars, trucks, buses, and motorcycles, are registered in this area [27].

### Study design and participants

We recruited motorcycle taxi drivers in six central provinces of Thailand between January to March 2023. The total number of motorcycle taxi drivers in the study areas was estimated at 135,231 [24]. The total sample size of participants was determined to be 383 using the following Equation (1)


$$\begin{array}{c} n=\frac{p(\text{1}-p)}{\frac{e^{\text{2}}}{Z^{\text{2}}}+\frac{p(\text{1}-p)}{N}} \end{array}$$ (1)

where n is the sample size, N=135,231 is the population size, e=0.05 is the level of precision, p=0.5 is the estimated proportion of the attribute of interest, Z=1.96 is the 97.5 percentile of Z-score of the standard normal distribution [28]. The sample size of participants in each province was determined in a proportionate stratified sampling, multiplying the total sample size by the number of motorcycle taxi drivers in the province divided by the total number of motorcycle taxi drivers in six provinces. The number of participants is shown in Table 1.

**Table 1:** The numbers and sample sizes of motorcycle taxi drivers.

Province	Number of Motorcycle Taxi Drivers^1^	Calculated Sample Size	Location	Obtained Sample Size
Bangkok (BKK)	84,889	240	Din Daeng Subdistrict, Din Daeng District	240
Nonthaburi (NBI)	10,346	29	Bang Phut Subdistrict, Pakkret District	34
Pathum Thani (PTT)	11,543	33	Khlong Nueng Subdistrict, Khlong Luang District	39
Samut Prakan (SPK)	17,218	49	Song Khanong Subdistrict, Phra Pradaeng District	55
Samut Sakhon (SKN)	4,949	14	Om Noi Subdistrict, Krathum Baen District	29
Nakhon Pathom (NPT)	6,286	18	Nakhon Pathom Subdistrict, Mueang District	44
Total	135,231	383		441

^1^source: [24].

### Data collection

#### Demographics, working characteristics, and symptoms.

Motorcycle taxi drivers were interviewed face-to-face using questionnaires before their lung function tests. The questionnaire included demographics, working characteristics, and respiratory symptoms (cough, phlegm, wheeze, and chest tightness). The questionnaire was designed based on ATS-DLD-78A by the American Thoracic Society and the investigation form by the Department of Disease Control, Ministry of Public Health, Thailand [29]. The participant’s height, weight, waist circumference, and blood pressure were measured on-site before lung function tests.

#### Personal air sampling (PAS).

Pollutants inhaled by motorcycle taxi drivers may be derived not only from motorcycle’s exhaust but also from the exhaust from other vehicles on the road, such as cars, trucks, and buses, as well as pollutants in ambient air. In order to measure the concentration of pollutants that may be inhaled by motorcycle taxi drivers, we installed the devices near their breathing zone. [30] and collected air samples during their working time. The personal exposures to PM_2.5_ and PM_10_ concentrations were measured by personal air sampling using a sampler (SKC, PCXR8, USA) operated at 2L/min, which was connected to a size-selective inlet for particulate matter with aerodynamic diameter ≤10 µm (PM_10_) or ≤2.5 µm (PM_2.5_) (SKC, Impact Sampler, USA) and polytetrafluoroethylene (PTFE) membrane filter (37-mm, 2µm pore size). The PM_10_ and PM_2.5_ filters were collected separately [30]. The reported values represent the mean of all individual samples (S4-S5 Table). All sampling durations were within the acceptable range as specified by the NIOSH Method 0600 and OSHA PV2121 protocols, ensuring compliance with established quality control standards. The mean sampling duration was 384 (minutes) with an SD of 39.9 (minutes) for PM_10_ and 393 (minutes) with an SD of 42.1 (minutes) for PM_2.5_. Smoking was not allowed during personal sampling. The samples were wrapped with aluminium foil and sealed in a zip-lock bag at the sampling site. They were transferred to the analytical lab and stored at −20 ◦C until being sent for analysis in order to prevent degradation. After collecting air samples, the humidity of the filters was absorbed in a desiccator by silica gel, and then the filters were passed through a static master (Mettler Toledo, Haug Deionizer) to eliminate the static electricity. Blank filters for the air samples were extracted and analyzed in the same protocol as the samples. Filters were weighed with microbalance (Mettler Toledo, XPR2U, Switzerland) at an average of three times, and the PM concentration of each sample was calculated using Equation (2).


$$\begin{array}{c} C=\frac{\left(W_{\text{2}}-W_{\text{1}}\right)-\left(B_{\text{2}}-B_{\text{1}}\right)}{V}\times {\text{10}}^{\text{3}} \end{array}$$ (2)

where C is the concentration (mg/m^3^), $W_{\text{1}}$ is the tare weight of the filter before sampling (mg), $W_{\text{2}}\;$ is the post-sampling weight of the sample-containing filter (mg), $B_{\text{1}}$ is mean tare weight of blank filters (mg), $B_{\text{2}}$ is the mean post-sampling weight of blank filters (mg), V is the sampled air volume (litre).

After measuring the PM_10_ and PM_2.5_ concentrations, PAHs were extracted from PM_10_ and PM_2.5_ filters according to the NIOSH method 5506 [31]. The filter was transferred to a glass extraction beaker with 5 mL of acetonitrile and placed in an ultrasonic bath for 30 minutes. A PTFE syringe filter with a pore size of 0.2 µm was used to collect the PAH solution. After adding 5 µm of dimethyl sulfoxide, the solution was evaporated with 99.99% nitrogen gas so that the sample volume became 50 µL. Then, the solution was transferred to an amber glass vial for the liquid chromatography analysis.

PAHs were quantified using a high-performance liquid chromatography instrument (Shimadzu, LC-40, Japan). Separation of the chemicals was performed in a 25 cm by 4.6 mm column, 5 µm particle size (Supelco Analytical, Supelcosil LC-PAH, USA). A mixture of water and acetonitrile (40:60) was used as the mobile phase. The chemicals were detected at a wavelength of 254 nm. The flow rate was 1.6 mL/min, and the injection volume was 25 µl. External calibration using mixed PAH standards was performed at seven calibration points. Calibration curves of 15 chemicals were linearly fitted with mean correlation coefficients greater than 0.9980. The percent of recovery ranged from 97.0 to 128.0 for all the chemicals in the internal standard. Limits of detection (LODs) was 0.05 mg/L.

This study measured the following 15 chemicals of PM-bound PAH: naphthalene, acenaphthene, fluorene, phenanthrene, anthracene, fluoranthene, pyrene, benzo (a)anthracene, chrysene, benzo(b)fluoranthene, benzo(k)fluoranthene, benzo(a)pyrene, dibenzo(ah)anthracene, benzo(ghi)perylene, and indeno(123-cd) pyrene. The concentration of total PAH was calculated from Equation (3).


$$\begin{array}{c} Total\;PAH=\;\sum_{i=\text{1}}^{\text{1}\text{5}}PAH_{i} \end{array}$$ (3)

where $PAH_{i}$ is the concentration of the i-th chemical (1≤i≤15).

Motorcycle taxi drivers from the same province were considered to belong to similar exposure groups (SEGs). The number of required samples for personal air sampling in each SEG was determined from the number of participants in the SEG according to the method described in the occupational exposure sampling strategy manual [32]. Table 2 shows the number of air samples required for and obtained by personal air sampling for PM_10_ and PM_2.5_. For each SEG, participants were selected randomly. Driving distance and the number of passengers on the day of personal air sampling were recorded. Owing to constraints in equipment availability and field logistics, not all participants were monitored for both PM_10_ and PM_2.5_. Personal air sampling was performed using separate devices for each particulate size fraction, and each participant was assigned only one sampler per sampling session. Consequently, the total number of samples collected differed slightly between PM_10_ (n = 151) and PM_2.5_ (n = 153). Each sample corresponds to an individual participant and represents exposure over a single work shift. No composite or pooled samples were collected across multiple individuals. In our study, a total of 22 workstations across six provinces were included (S2 Table and S9-S10 Figs). At each workstation, motorcycle taxi drivers were randomly assigned to wear either a PM₁₀ or a PM₂.₅ personal air sampler (one device per individual) throughout their working hours while transporting passengers (S11 Fig). Each pollutant was thus measured independently on separate subjects, with each driver representing a distinct exposure under actual occupational conditions. A key limitation of the study, as rightly noted, is the absence of GPS tracking or detailed route information. Given the nature of their occupation, motorcycle taxi drivers in Thailand travel along variable routes that are influenced by passenger destinations and daily demand patterns. Consequently, individual exposure levels to PM₁₀ and PM₂.₅ may have been affected by route-specific environmental conditions, which could contribute to variability in the concentration data. For future studies, incorporating GPS tracking or route logging would facilitate more precise spatiotemporal assessments of exposure and strengthen the ability to link measured pollution levels to specific geographic locations and environmental conditions.

**Table 2:** The number of personal air samples of motorcycle taxi drivers.

Province	Number of participants in SEG	Number of required air samples	Number of obtained air samples in SEG	
			PM_10_	PM_2.5_
Bangkok	240	22	47	50
Nonthaburi	34	16	23	18
Pathum Thani	39	17	22	25
Samut Prakan	55	18	27	24
Samut Sakhon	29	15	16	19
Nakhon Prathom	44	17	16	17
Total	441	105	151	153

#### Incremental lifetime cancer risk (ILCR).

The International Agency for Research on Cancer classified PAH chemicals into five groups. Group 1 is a chemical carcinogenic to humans (benzo(a) pyrene), group 2A is a chemical probably carcinogenic to humans (dibenzo(a,h) anthracene), group 2B consists of chemicals possibly carcinogenic to humans (naphthalene, benzo(a) anthracene, chrysene, benzo(b) fluoranthene, benzo(k) fluoranthene, indeno(123-c,d) pyrene), and group 3 consists of chemicals not classifiable as carcinogenic to humans (acenaphthene, fluorene, phenanthrene, anthracene, fluoranthene, pyrene, benzo(g,h,i) perylene) [33].

The incremental lifetime cancer risk (ILCR) represents the increased probability of the occurrence of tumorous diseases above the general average due to the impact of compounds suspected of having carcinogenic effects [34]. The total benzo(a) pyrene equivalent (BaP_eq_) concentration was calculated using the following equation:


$$\begin{array}{c} Total\;BaP_{eq}=\;\sum_{i=\text{1}}^{\text{1}\text{5}}C_{i}\times {TEF}_{i}, \end{array}$$ (4)

where $C_{i}$ is the concentration of the i-th chemical (mg/m^3^), ${TEF}_{i}$ is the toxic equivalency factor of an individual PAH, and 1≤i≤15 represents the index of 15 PAH chemicals. The value of $TEF_{i}$ was retrieved from Nisbet & LaGoy [35]. The $TEF_{i}$ of dibenzo(a,h) anthracene was set to 1.0 according to other studies [14,36]. The values of parameters in $TEF_{i}$ in Equation (4) are shown in S6 and S7 Tables.

The lifetime average daily dose (LADD) (mg/kg ∙ day) was calculated as


$$\begin{array}{c} LADD\;=\;\frac{Total\;BaP_{eq}\times IR\times ET\times EF\times ED}{BW\times AT\times H}, \end{array}$$ (5)

where IR=15.3 is air inhalation rate (m^3^/day) [37], ET is exposure time (hours/day), EF is exposure frequency (days/year), ED is exposure duration (years), BW is body weight (kg), AT=25,500 is averaging time for carcinogens (days) [38,39], and H=24 is hours in a day (hours/day).

The incremental lifetime cancer risk (ILCR) of the inhalation pathway was calculated by


$$\begin{array}{c} Incremental\;Lifetime\;Cancer\;Risk\;(ILCR)=LADD\times CSF \end{array}$$ (6)

where CSF=3.14 is inhalation cancer slope factor (mg/kg ∙ day) [38,39]. We calculated the LADD and ILCR using the mean values observed in each province for total BaPeq, ET, EF, ED, BW and shared values for IR and AT. These values are provided in S8 Table.

#### Area air sampling (AAS).

For each province, we set an optical particle counter (Aeroqual, AQM65, New Zealand) at a randomly selected workstation of motorcycle taxi drivers for area air sampling (AAS). The device contained cyclones for PM_10_ and PM_2.5_ and operated at 1 L/min. The average 24-hour PM_10_ and PM_2.5_ concentrations in ambient air were measured using the device during each province’s personal air sampling period. We could not collect area air sampling data from Samut Prakan due to a technical error.

#### Air quality monitoring station (AQM).

The average 24-hour of PM_10_ and PM_2.5_ concentrations in ambient air at air quality monitoring (AQM) stations in Bangkok, Nonthaburi, Pathum Thani, Samut Prakan, Samut Sakhon, and Nakhonpathom from January 1, 2023, to March 31, 2023, were retrieved from a database of the Pollution Control Department in Thailand [40].

#### Lung function tests.

For each participant, three lung function parameters, the forced vital capacity (FVC), the forced expiratory volume in 1 sec (FEV1), and the ratio of FEV1 to FVC (FEV1/FVC) were measured using spirometer (FIM MEDICAL, model Q13 SPIROLYSER, France) according to the ATS standard [41–43]. A set of predicted equations for Thai adults [44,45] was used to calculate percent predicted values of FVC, FEV1, FEV1/FVC (%predicted FVC, %predicted FEV1, and %predicted FEV1/FVC) and Lower Limit of Normal FVC, FEV1, and FEV1/FVC (LLN-FVC, LLN-FEV1, LLN-FEV1/FVC). The lung function test results were categorized into four types: normal, restrictive, obstructive, and combined (Table 3) [46].

**Table 3:** Classification of lung function test results.

Lung function test result	FEV₁/FVC	FVC	Interpretation Criteria
Normal	≥ LLN-FEV1/FVC	≥ LLN-FVC	No obstruction or restriction
Restrictive	≥ LLN-FEV1/FVC	<LLN-FVC	Restricted lung volume
Obstructive	<LLN-FEV1/FVC	≥ LLN-FVC	Airflow obstruction without volume loss
Combined	<LLN-FEV1-FVC	<LLN-FVC	Airflow obstruction with restrictive pattern

### Statistical tests

Descriptive statistics, including percentage, median, mean, standard deviation (S.D.), minimum (min.), and maximum (max.), were used to describe participants’ demographics, PM concentrations, PM-bound total PAHs concentration, and lung function parameters. Pearson’s correlation coefficients were used to analyze the association between two numerical variables. Spearman’s correlation coefficients were used instead when data contained an obvious pair of outliers. The difference in a numerical parameter between two groups was analyzed by t-test, and the difference among more than two groups was analyzed by one-way ANOVA. Fisher’s exact test was used to analyze contingency tables of symptoms. All statistical analyses were performed using R studio (version 2023.12.0). P-values less than 0.05 were considered as significant.

### Ethical approval

Ethical approval for this study was granted by the Human Research Ethics Committee of Thammasat University, No. 3 (Approval No. 031/2565), on April 28, 2024. Prior to data collection, written informed consent was obtained from all participants. Participants were clearly informed about the study’s objectives, procedures, potential risks, and benefits. They were also assured of the confidentiality and anonymity of their responses and given the opportunity to ask questions before signing the consent form. All data were fully anonymized before being accessed by the research team. No minors were included in this study, and no waiver of consent was granted by the ethics committee.

## Results and discussion

### Demographics, working characteristics, and symptoms

S3 Table shows the demographics and working characteristics of 441 participants recruited in six provinces in this study. More than 90% of participants were male, and about 60% were between 40 and 59 years old. Current smokers occupied 44.2%, 66.0% had no physical exercise, and 47.8% had normal body mass index. Most participants (92.3%) worked more than 8 hours daily, and 70.5% wore surgical masks (70.5%). Almost half (49.7%) of participants had more than ten years of work experience.

Table 4 shows the disease symptoms answered in questionnaires by participants. Pathum Thani (PTT) had the highest proportion of participants who answered symptoms of chronic bronchitis (28.2%), followed by Samut Prakan (SPK) (12.7%) and Bangkok (BKK) (10.4%). The participants in Pathum Thani also had the highest proportion of participants with symptoms of acute bronchitis (17.9%), followed by Samut Prakan (3.6%) and Bangkok (2.9%). It is important to clarify that these respiratory conditions were identified based on participants’ responses to structured questionnaires rather than through clinical diagnosis or medical record verification. As such, the reported symptoms represent subjective health status rather than physician-confirmed diseases. Although self-reported symptom data are subject to inherent limitations, including reporting bias and limited diagnostic accuracy, they are widely utilized in occupational and environmental epidemiology as a practical and cost-effective method for conducting preliminary health assessments in large population-based studies. In the present study, respiratory symptoms such as those indicative of chronic and acute bronchitis were obtained through participant self-reports, offering initial insights into potential associations between PM-bound PAH exposure and respiratory health outcomes across provinces.

**Table 4:** Disease symptoms answered in the questionnaire (n = 441).

Characteristics	BKK*	NBI*	PTT*	SPK*	SKN*	NPT*	Total (%)
Acute bronchitis							
Yes	7 (2.9)	1 (2.9)	7 (17.9)	2 (3.6)	1 (3.4)	1 (2.3)	19 (4.3)
No	233 (97.1)	33 (97.1)	32 (82.1)	53 (96.4)	28 (96.6)	43 (97.7)	422 (95.7)
Chronic bronchitis							
Yes	25 (10.4)	2 (5.9)	11 (28.2)	7 (12.7)	1 (3.4)	2 (4.5)	48 (10.9)
No	215 (89.6)	32 (94.1)	28 (71.8)	48 (87.3)	28 (96.6)	42 (95.5)	393 (89.1)
Bronchial Asthma							
Yes	4 (1.7)	0 (0.0)	1 (2.6)	0 (0.0)	0 (0.0)	1 (2.3)	6 (1.4)
No	236 (98.3)	34 (100.0)	38 (97.4)	55 (100.0)	29 (100.0)	43 (97.7)	435 (98.6)
Chronic Obstructive Pulmonary Disease							
Yes	3 (1.2)	0 (0.0)	1 (2.6)	0 (0.0)	0 (0.0)	1 (2.3)	5 (1.1)
No	237 (98.8)	34 (100.0)	38 (97.4)	55 (100.0)	29 (100.0)	43 (97.7)	436 (98.9)
Persistent cough							
Yes	3 (1.2)	0 (0.0)	0 (0.0)	1 (1.8)	1 (3.4)	0 (0.0)	5 (1.1)
No	235 (98.8)	34 (100.0)	39 (100.0)	54 (98.2)	29 (96.6)	44 (100.0)	436 (98.9)
Persistent phlegm							
Yes	5 (2.1)	0 (0.0)	0 (0.0)	1 (1.8)	0 (0.0)	0 (0.0)	6 (1.4)
No	235 (97.9)	34 (100.0)	39 (100.0)	54 (98.2)	29 (100.0)	44 (100.0)	435 (98.6)
Nasal allergy							
Yes	13 (5.4)	1 (2.9)	1 (2.6)	2 (3.6)	0 (0.0)	1 (2.3)	18 (4.1)
No	227 (94.6)	33 (97.1)	38 (97.4)	53 (96.4)	29 (100.0)	43 (97.7)	423 (95.9)
Diabetes							
Yes	18 (7.5)	8 (23.5)	5 (12.8)	10 (18.2)	6 (20.7)	4 (9.1)	51 (11.6)
No	222 (92.5)	26 (76.5)	34 (87.2)	45 (81.8)	23 (79.3)	40 (90.9)	390 (88.4)
Hypertension							
Yes	47 (19.6)	9 (26.5)	11 (28.2)	13 (23.6)	5 (17.2)	12 (27.3)	97 (22.0)
No	193 (80.4)	25 (73.5)	28 (71.8)	42 (76.4)	24 (82.8)	32 (72.7)	344 (78.0)
Allergy skin rash							
Yes	2 (0.8)	0 (0.0)	1 (2.6)	3 (5.5)	1 (3.4)	0 (0.0)	7 (1.6)
No	238 (99.2)	34 (100.0)	38 (97.4)	52 (94.5)	28 (96.6)	44 (100.0)	434 (98.4)
Old tuberculosis							
Yes	2 (0.8)	0 (0.0)	0 (0.0)	0 (0.0)	0 (0.0)	0 (0.0)	2 (0.5)
No	238 (99.2)	34 (100.0)	39 (100.0)	55 (100.0)	29 (100.0)	44 (100.0)	439 (99.5)
Neuromuscular							
Yes	6 (2.5)	0 (0.0)	3 (7.7)	1 (1.8)	0 (0.0)	0 (0.0)	10 (2.3)
No	234 (97.5)	34 (100.0)	36 (92.3)	54 (98.2)	29 (100.0)	44 (100.0)	431 (97.7)
Chest pain							
Yes	3 (1.2)	1 (2.9)	2 (5.1)	6 (10.9)	0 (0.0)	1 (2.3)	13 (2.9)
No	237 (98.8)	33 (97.1)	37 (94.9)	49 (89.1)	29 (100.0)	43 (97.7)	428 (97.1)
History of allergy							
Yes	8 (3.3)	2 (5.9)	1 (2.6)	7 (12.7)	1 (3.4)	2 (4.5)	21 (4.8)
No	232 (96.7)	32 (94.1)	38 (97.4)	48 (87.3)	28 (96.6)	42 (95.5)	420 (95.2)
History of asthma							
Yes	9 (3.8)	0 (0.0)	3 (7.7)	3 (5.5)	1 (3.4)	1 (2.3)	17 (3.9)
No	231 (96.2)	34 (100.0)	36 (92.3)	52 (94.5)	28 (96.6)	43 (97.7)	424 (96.1)
History of pneumonia							
Yes	0 (0.0)	0 (0.0)	0 (0.0)	1 (1.8)	0 (0.0)	0 (0.0)	1 (0.2)
No	240 (100.0)	34 (100.0)	39 (100.0)	54 (98.2)	29 (100.0)	44 (100.0)	440 (99.8)

*Bangkok (BKK), Nonthaburi (NBI), Pathum Thani (PTT), Samut Prakan (SPK), Samut Sakhon (SKN), and Nakhon Pathom (NPT).

### Personal exposure levels of PM and PM-bound PAHs

#### Concentrations of PM and PM-bound PAH.

S4 Table shows the concentration of particulate matter with aerodynamic diameter ≤10 µm (PM_10_) and PM_10−_bound total PAHs measured in personal air sampling in each province. The mean of PM_10_ concentration was highest in Pathum Thani (224.9 µg/m^3^), followed by Nakhon Pathom (169.6 µg/m^3^) and Samut Sakhon (165.8 µg/m^3^). The mean of PM_10−_bound total PAH concentration was highest in Pathum Thani (38.4 ng/m^3^), followed by Bangkok (11.1 ng/m^3^) and Samut Prakan (2.9 ng/m^3^).

S5 Table shows the concentration of particulate matter with aerodynamic diameter ≤2.5 µm (PM_2.5_) and PM_2.5_-bound total PAHs from personal air sampling. The mean of PM_2.5_ concentration was highest in Pathum Thani (410.9 µg/m^3^), followed by Nonthaburi (206.2 µg/m^3^) and Bangkok (132.2 µg/m^3^). The mean of PM_2.5_-bound total PAH concentration was highest in Pathum Thani (36.9 ng/m^3^), followed by Bangkok (6.7 ng/m^3^) and Samut Prakan (3.3 ng/m^3^). In Pathum Thani, the mean PM₂.₅ concentration (410.9 μg/m³) was higher than that of PM₁₀ (224.9 μg/m³), and a similar pattern was observed in Nonthaburi, where the mean PM₂.₅ concentration (206.2 μg/m³) exceeded the mean PM₁₀ concentration (162.6 μg/m³). This discrepancy is likely attributable to the non-simultaneous collection of PM₁₀ and PM₂.₅ samples from different motorcycle taxi drivers, which may have introduced temporal and spatial variability in exposure measurements.

The Pathum Thani had the highest means of PM_10_ concentrations, PM_10_-bound total PAHs (S4 Table), PM_2.5_ concentration, and PM_2.5_-bound total PAHs (S5 Table) in six provinces. The composition of PM_10_-bound PAH and PM_2.5_-bound PAH chemicals are provided in S9 and S10 Tables, respectively.

The observed phenomenon where mass concentrations of PM_2.5_ are nearly twice those of PM₁₀ and can be attributed to the predominance of fine particulate matter in urban traffic environments, where combustion-related particles are abundant. PM₂.₅ often originates from vehicle exhaust and secondary aerosol formation, which tend to dominate over coarser particles (PM₁₀ minus PM₂.₅ fraction) such as road dust. Conversely, PM₁₀-bound PAH concentrations are higher than those of PM₂.₅-bound PAHs may reflect the association of certain PAH compounds with larger particles, which can adsorb more PAHs due to greater surface area or different chemical affinities. Furthermore, each motorcycle taxi driver’s profile is influenced by their unique route for passenger pick-up and drop-off, resulting in spatial variability in particulate and PAH exposure. This variability causes the PM and PM-bound PAH concentrations measured personally to differ according to specific microenvironments encountered. Consequently, the PM₁₀-bound and PM₂.₅-bound total PAHs are likely derived from distinct sources or source mixes, reflecting the heterogeneity in emission profiles along different routes.

**Fig 1:**
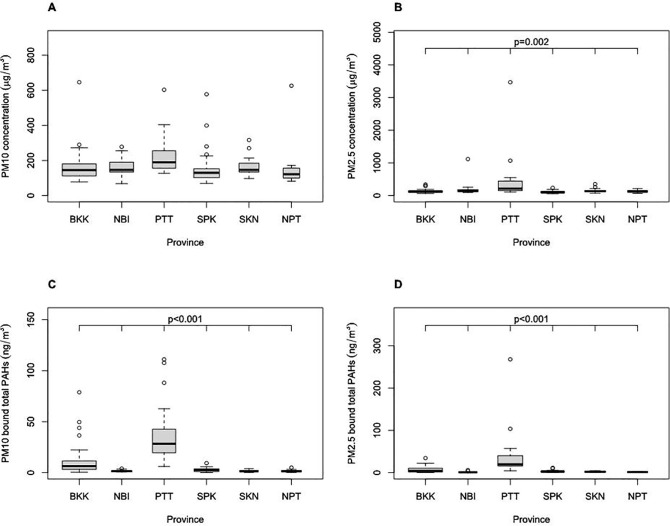
Particulate matter concentrations measured by personal air sampling in six provinces: Bangkok (BKK), Nonthaburi (NBI), Pathum Thani (PTT), Samut Prakan (SPK), Samut Sakhon (SKN), and Nakhon Pathom (NPT). (A) PM_10_; (B) PM_2.5_; (C) PM_10_ bound total PAHs; (D) PM_2.5_ bound total PAHs. P-values were shown if means of concentrations from six provinces were shown to be significantly different by one-way ANOVA test.

Fig 1 shows the particulate matter concentrations measured by personal air sampling (PAS) from six provinces. The mean of PM_2.5_ concentrations differed depending on province (p = 0.002 with one-way ANOVA). The mean of PM_10_-bound total PAHs differed depending on province (p < 0.001 with one-way ANOVA). The mean of PM_2.5_-bound total PAHs differed depending on province (p < 0.001 with one-way ANOVA). In the PM_10_ concentrations, the correlation was not significant. These results indicated that the exposure of PM_2.5_ concentrations, PM_10_-bound total PAHs, and PM_2.5_-bound total PAHs were associated with the province where motorcycle taxi drivers were services. Prior to conducting one-way ANOVA, the assumptions of normality and homogeneity of variances were evaluated. The Shapiro–Wilk test was used to assess the normality of distribution, while Levene’s test was employed to examine the equality of variances across groups. These assumptions were met for PM₂.₅, PM₁₀-bound total PAHs, and PM₂.₅-bound total PAHs, supporting the use of one-way ANOVA for these parameters. Following the ANOVA, post hoc pairwise comparisons were conducted using Tukey’s Honestly Significant Difference (HSD) test to identify statistically significant differences between provinces. In the case of PM₁₀, although measurements were available for all six provinces, the interprovincial variation in mean concentrations was not statistically significant, and the data did not fully satisfy the assumptions required for parametric testing. Consequently, neither ANOVA nor post hoc tests were applied to PM₁₀ data.

#### Relationship between concentration measured by PAS and AQM or AAS.

S1 Fig shows the relationship between PM concentrations measured by air quality monitoring station (AQM) and area air sampling (AAS). There were significant correlations between the daily PM concentrations collected by AQM and AAS for both PM_10_ (S1(A) Fig) and PM_2.5_ (S1(B) Fig). Although significant correlations were observed between AQM and AAS data, the two approaches capture different aspects of PM exposure. AQM stations typically provide continuous 24-hour average measurements that reflect broader ambient background levels, often at elevated or centralized locations. In contrast, AAS offers localized, short-duration measurements that are highly sensitive to site-specific conditions and immediate surroundings. Consequently, absolute concentration values may differ substantially between methods, despite consistent temporal or spatial trends. These methodological differences highlight the complementary nature of AQM and AAS in providing a comprehensive assessment of environmental PM exposure. S2 Fig shows the relationship between PM concentrations measured by personal air sampling (PAS) and AQM and the relationship between PM concentrations measured by PAS and AAS. PM concentrations collected by PAS were not significantly correlated with PM concentrations measured by AQM or AAS for both PM_10_ and PM_2.5_ (S2(A)(B)(C)(D) Fig). These results indicated that the PM_10_ and PM_2.5_ concentrations measured by PAS are independent of values monitored at AQMs or AASs. Therefore, the PM data measured by PAS cannot be replaced by those in AQM or AAS. These results highlighted the importance of pollution data measured by PAS in this study.

**Fig 2:**
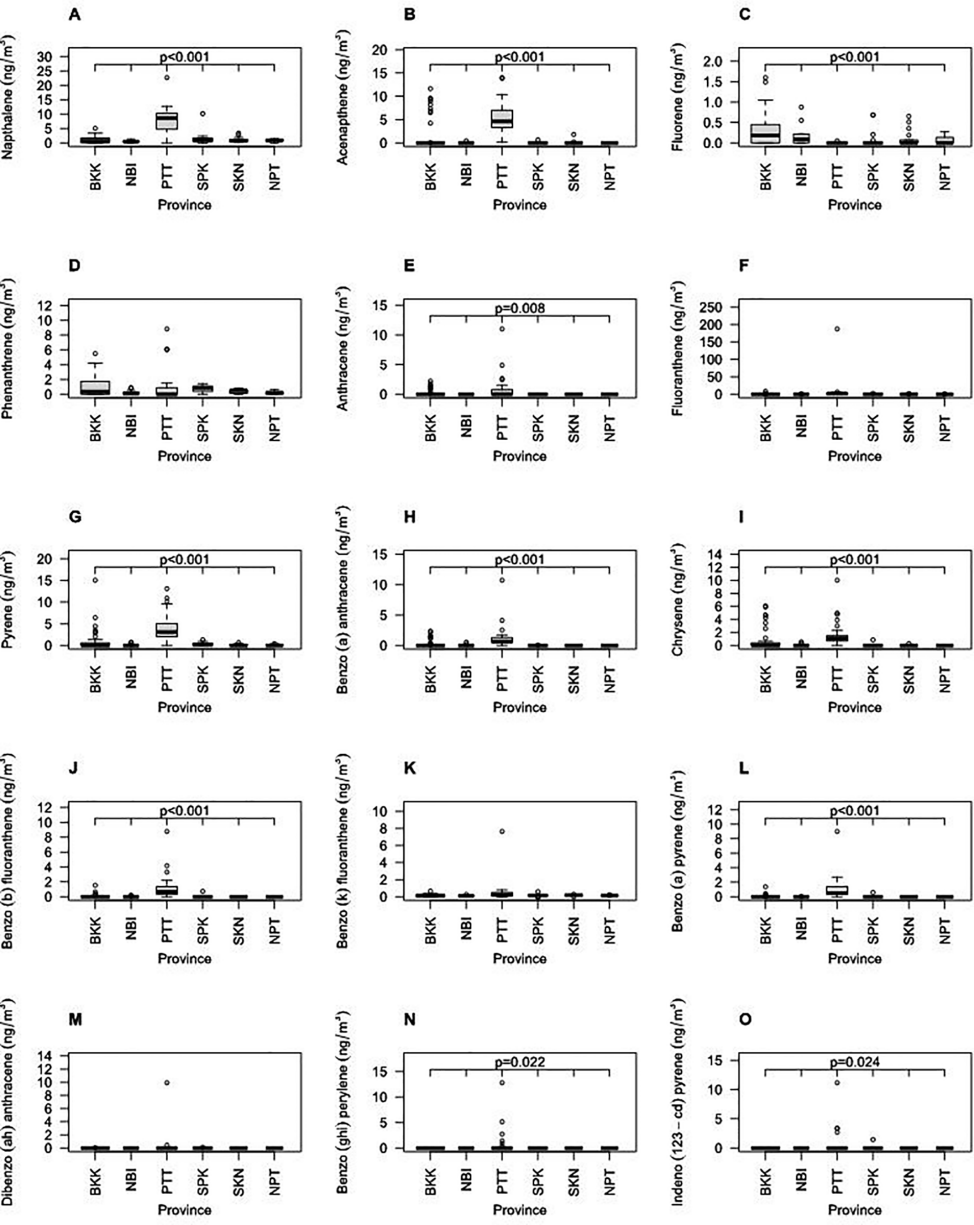
PM_2.5_ bound PAHs measured by personal air sampling from six provinces. Bangkok (BKK), Nonthaburi (NBI), Pathum Thani (PTT), Samut Prakan (SPK), Samut Sakhon (SKN), and Nakhon Pathom (NPT). (A) Napthalene; (B) Acenapthene; (C) Fluorene; (D) Phenanthrene; (E) Anthracene; (F) Fluoranthene; (G) Pyrene; (H) Benzo(a) anthracene; (I) Chrysene; (J) Benzo(b) fluoranthene; (K) Benzo(k) fluoranthene; (L) Benzo(a) pyrene; (M) Dibenzo(a,h) anthracene; (N) Benzo(g,h,i) perylene; (O) Indeno(123-c,d) pyrene. P-values were shown if means of concentrations from six provinces were shown to be significant different by one-way ANOVA test.

Fig 2 shows the concentrations of PM_2.5_-bound PAH chemicals measured by PAS from six provinces. Some of PM_2.5_-bound PAHs chemical concentrations differed depending on the province, namely naphthalene (p < 0.001), Acenapthene (p < 0.001), fluorene (p < 0.001), anthracene (p = 0.008), Pyrene (p < 0.001), Benzo(a) anthracene (p < 0.001), Chrysene (p < 0.001), Benzo(b) fluoranthene (p < 0.001), Benzo(a) pyrene (p < 0.001), Benzo(g,h,i) perylene (p = 0.022), and Indeno(123-c,d) pyrene (p = 0.024). These differences likely reflect variations in local emission sources, source strengths, and combustion conditions. Lighter PAHs, such as naphthalene and fluorene, are commonly associated with low-temperature combustion processes (e.g., gasoline-powered engines), while heavier PAHs, including benzo(a)pyrene and chrysene, are typically emitted from high-temperature combustion sources (e.g., diesel engines, industrial emissions). The variation in PAH profiles may thus correspond to differences in traffic composition, industrial activities, and urban density across provinces. Additionally, meteorological factors such as wind speed, atmospheric stability, and temperature can influence the dispersion and accumulation of PAHs, contributing to the observed disparities. Conversely, the absence of significant variation in certain PAHs may suggest more regionally consistent emission sources or background levels less influenced by local activities. These findings highlight the complex and heterogeneous nature of PAH exposure and underscore the need for further source apportionment analysis to better characterize the origin and distribution of PAHs in different geographic areas.

S4 Fig shows the concentrations of PM_10−_bound PAH chemicals measured by PAS from six provinces. Some PM_10−_bound PAHs chemical concentrations differed depending on the province, namely naphthalene (p < 0.001), Acenapthene (p < 0.001), fluorene (p < 0.001), phenanthrene (p < 0.001), anthracene (p < 0.001), fluoranthene (p < 0.001), Pyrene (p < 0.001), Benzo(a) anthracene (p < 0.001), Benzo(b) fluoranthene (p < 0.001), Benzo(k) fluoranthene (p = 0.004), Benzo(a) pyrene (p = 0.008), and Benzo(g,h,i) perylene (p < 0.001). These results indicated that motorcycle taxi drivers from different provinces were exposed to different amounts of PM_10−_bound PAHs chemicals.

#### Relationship between PM-bound total PAH concentrations and bronchitis symptoms.

**Fig 3:**
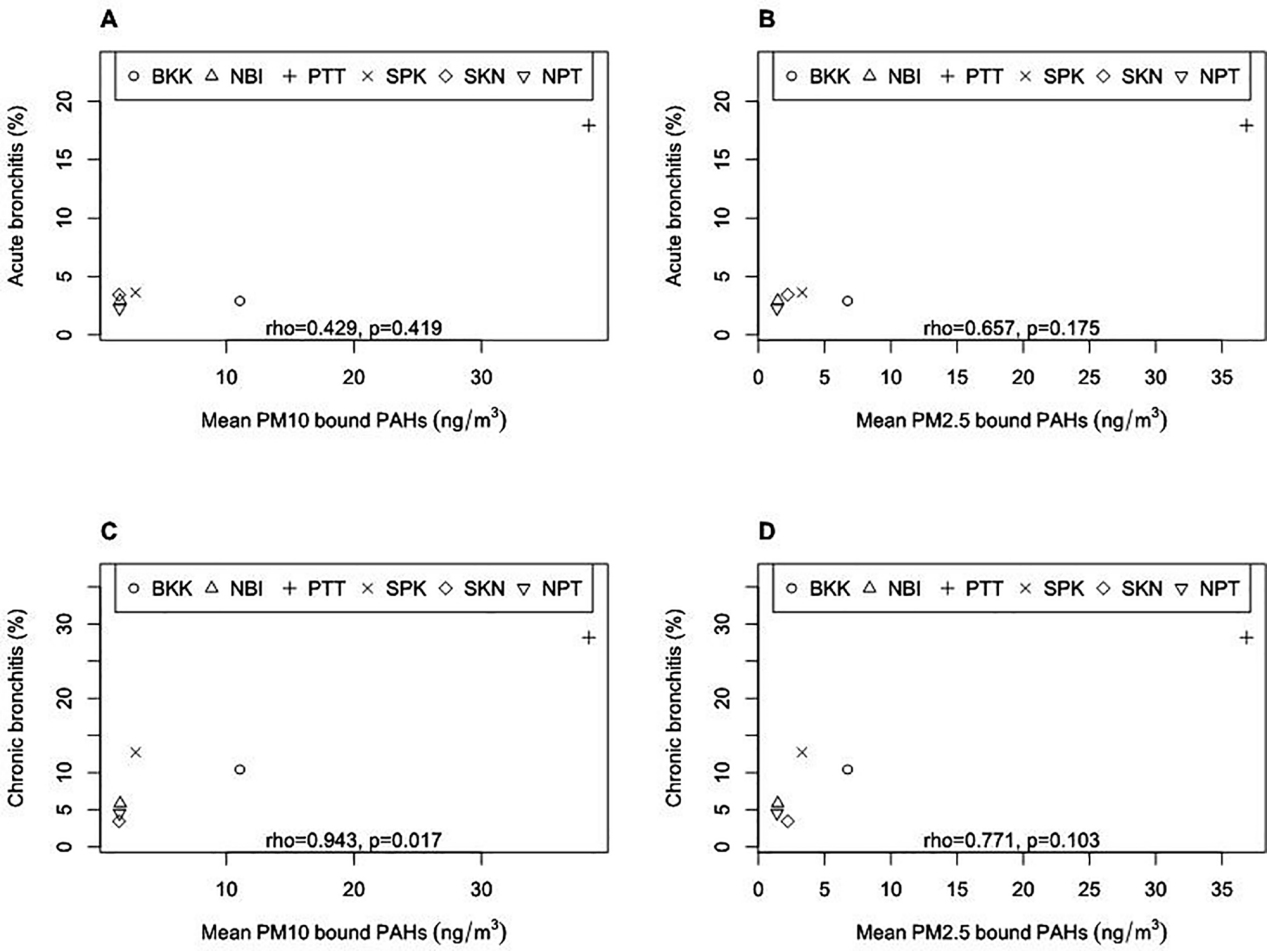
The relationship between the percentage of participants who answered symptoms of acute or chronic bronchitis in their questionnaires and the mean of PM concentrations measured by personal air sampling in each province. Bangkok (BKK), Nonthaburi (NBI), Pathum Thani (PTT), Samut Prakan (SPK), Samut Sakhon (SKN), and Nakhon Pathom (NPT). (A) acute bronchitis and PM_10−_bound total PAHs; (B) acute bronchitis and PM_2.5_-bound total PAHs; (C) chronic bronchitis and PM_10−_bound total PAHs; (D) chronic bronchitis and PM_2.5_-bound total PAHs. Spearman’s correlation coefficient and the p-value were shown.

Fig 3 shows the relationship between the percentage of participants who answered symptoms in their questionnaires and the mean of PM_10−_bound total PAHs and PM_2.5_-bound total PAHs measured by personal air sampling in each province. The proportion of participants reporting chronic bronchitis symptoms at the provincial level demonstrated a strong positive correlation with the province’s mean PM₁₀-bound total PAH concentrations (Spearman’s correlation coefficient = 0.943, p = 0.017). In contrast, no statistically significant association was observed between self-reported chronic bronchitis symptoms and individual-level PM₁₀-bound total PAH exposure. This finding suggests that while a province-level relationship is evident, it does not necessarily translate to consistent associations at the individual level. Such discrepancies may be attributed to variability in individual susceptibility, exposure misclassification, or other unmeasured confounders (S6 Fig). Assuming that motorcycle taxi drivers in the same province were exposed to a similar amount of PM and PM-bound PAH, these results indicated that motorcycle taxi drivers’ exposure to PM_10_-bound PAH was associated with chronic bronchitis symptoms.

#### Incremental lifetime cancer risk.

Table 5 shows the total benzo(a) pyrene equivalent (BaP_eq_) concentrations, the lifetime average daily dose (LADD) and the incremental lifetime cancer risk (ILCR). The highest total BaP_eq_ of PM_2.5_-bound PAH was observed in Pathum Thani (2.0 × 10^−6^), followed by Bangkok (1.2 × 10^−7^) and Samut Prakan (6.1 × 10^−8^). The highest LADD of PM_2.5_-bound PAH was observed in Pathum Thani (2.5 × 10^−8^ mg/kg ∙ day), followed by Bangkok (1.8 × 10^−9^ mg/kg ∙ day) and Samut Prakan (9.9 × 10^−10^ mg/kg ∙ day). The highest ILCR of PM_2.5_-bound PAH was estimated in Pathum Thani (7.8 × 10^−8^), followed by Bangkok (5.8 × 10^−9^) and Samut Prakan (3.1 × 10^−9^). The lifetime cancer risk of benzo(a) pyrene was categorized into three levels: less than 1 × 10^−6^ indicates an acceptable risk, between 1 × 10^−6^ to 1 × 10^−4^ indicates a potential risk, and greater than 1 × 10^−4^ indicates a public health concern that requires urgent action [16,47–50]. Our result indicated that motorcycle taxi drivers in all six provinces had an acceptable risk level of incremental lifetime cancer risk from PM_2.5_-bound PAH and PM_10−_bound PAH.

**Table 5:** The incremental lifetime cancer risk (ILCR) of PM-bound PAH in six provinces.

Province	PM_10 − _bound PAH			PM_2.5_-bound PAH		
	total BaP_eq_	LADD	ILCR	total BaP_eq_	LADD	ILCR
Bangkok	4.1 × 10^ − 7^	6.1 × 10^ − 9^	1.9 × 10^ − 8^	1.2 × 10^ − 7^	1.8 × 10^ − 9^	5.8 × 10^ − 9^
Nonthaburi	2.6 × 10^ − 8^	3.8 × 10^ − 10^	1.2 × 10^ − 9^	2.8 × 10^ − 8^	4.0 × 10^ − 10^	1.2 × 10^ − 9^
Pathum Thani	1.2 × 10^ − 6^	1.4 × 10^ − 8^	4.5 × 10^ − 8^	2.0 × 10^ − 6^	2.5 × 10^ − 8^	7.8 × 10^ − 8^
Samut Prakan	6.7 × 10^ − 8^	1.1 × 10^ − 9^	3.4 × 10^ − 9^	6.1 × 10^ − 8^	9.9 × 10^ − 10^	3.1 × 10^ − 9^
Samut Sakhon	2.5 × 10^ − 8^	2.9 × 10^ − 10^	9.2 × 10^ − 10^	2.0 × 10^ − 8^	2.3 × 10^ − 10^	7.3 × 10^ − 10^
Nakhon Prathom	1.7 × 10^ − 8^	3.0 × 10^ − 10^	9.3 × 10^ − 10^	1.7 × 10^ − 8^	2.9 × 10^ − 10^	9.0 × 10^ − 10^

### Lung function test

#### Statistics of lung function parameters in six provinces.

Table 6 shows statistics of lung function parameters of participants in six provinces. Percent predicted FVCs of all participants had a mean of 89.57 with SD of 17.82. The mean of percent predicted FVC was lowest in Nonthaburi (81.88%), followed by Bangkok (86.77%) and Samut Prakan (92.59%). The means of percent predicted FVC were significantly different among six provinces (p < 0.001) (S11 Table). Percent predicted FEV1s had 96.60 with SD of 17.63. There was no significant difference in the means of percent predicted FEV1 among six provinces (S12 Table). Percent predicted FEV1/FVCs had 104.29 with SD of 11.80. The mean percent predicted FEV1/FVC was the lowest in Nakhon Prathom (95.93%), followed by Pathum Thani (98.34%) and Samut Prakan (99.70%). The means of percent predicted FEV1/FVCs were significantly different among six provinces (p < 0.001) (S13 Table).

**Table 6:** Statistics of lung function parameters of participants in six provinces.

Parameters	Province	n	Median	Mean	SD	Min	Max
FVC (%predicted)	Bangkok	240	86.24	86.77	15.49	45.68	131.80
	Nonthaburi	34	81.69	81.88	14.17	54.26	117.41
	Pathum Thani	39	93.08	93.33	21.22	42.51	148.28
	Samut Prakan	55	93.91	92.59	16.72	50.38	129.18
	Samut Sakhon	29	101.93	98.38	18.65	61.46	146.49
	Nakhon Prathom	44	97.60	97.90	23.41	49.33	151.23
	Total	441	89.55	89.57	17.82	42.51	151.23
FEV1 (%predicted)	Bangkok	240	98.02	96.56	16.81	37.28	146.98
	Nonthaburi	34	94.88	96.37	16.41	68.34	134.95
	Pathum Thani	39	96.72	95.00	21.18	37.93	141.39
	Samut Prakan	55	97.26	95.94	18.61	40.80	145.58
	Samut Sakhon	29	102.99	102.46	19.49	54.41	132.12
	Nakhon Prathom	44	95.41	95.36	17.24	50.71	125.40
	Total	441	97.84	96.60	17.63	37.28	146.98
FEV1/FVC (%predicted)	Bangkok	240	107.5	107.12	9.89	57.08	125.07
	Nonthaburi	34	113.00	113.11	6.84	96.14	128.92
	Pathum Thani	39	100.86	98.34	13.44	50.40	125.17
	Samut Prakan	55	101.37	99.70	11.13	64.60	120.13
	Samut Sakhon	29	101.5	99.86	10.78	80.66	125.94
	Nakhon Prathom	44	99.34	95.93	14.30	57.57	120.20
	Total	441	105.02	104.29	11.80	50.40	128.92

Table 7 shows statistics of lung function abnormality suggested by the percent predicted FVC and percent predicted FEV1/FVC. The participants in six provinces had an abnormal pattern of 154 (34.9%). The Nonthaburi had the highest abnormal among the six provinces. Of 17 participants in Nonthaburi (50.0%) showed the restrictive pattern.

**Table 7:** Lung function abnormality suggested by percent predicted FVC and percent predicted FEV1/FVC.

Province	n	Normal (%)	Abnormal (%)	Type of abnormal		
				Restrictive (%)	Obstructive (%)	Combined (%)
Bangkok	240	163 (67.9)	77 (32.1)	69 (28.8)	2 (0.8)	6 (0.8)
Nonthaburi	34	17 (50.0)	17 (50.0)	17 (50.0)	0 (0.0)	0 (0.0)
Pathum Thani	39	25 (64.1)	14 (35.9)	5 (12.8)	6 (15.4)	3 (15.4)
Samut Prakan	55	39 (70.9)	16 (29.1)	8 (14.5)	6 (10.9)	2 (10.9)
Samut Sakhon	29	19 (65.5)	10 (34.5)	4 (13.8)	5 (17.2)	1 (17.2)
Nakhon Prathom	44	24 (54.5)	20 (45.5)	9 (20.5)	10 (22.7)	1 (22.7)
Total	441	287 (65.1)	154 (34.9)	112 (25.4)	29 (6.6)	13 (6.6)

#### Lung function parameters and symptoms of bronchitis answered in questionnaires.

**Fig 4:**
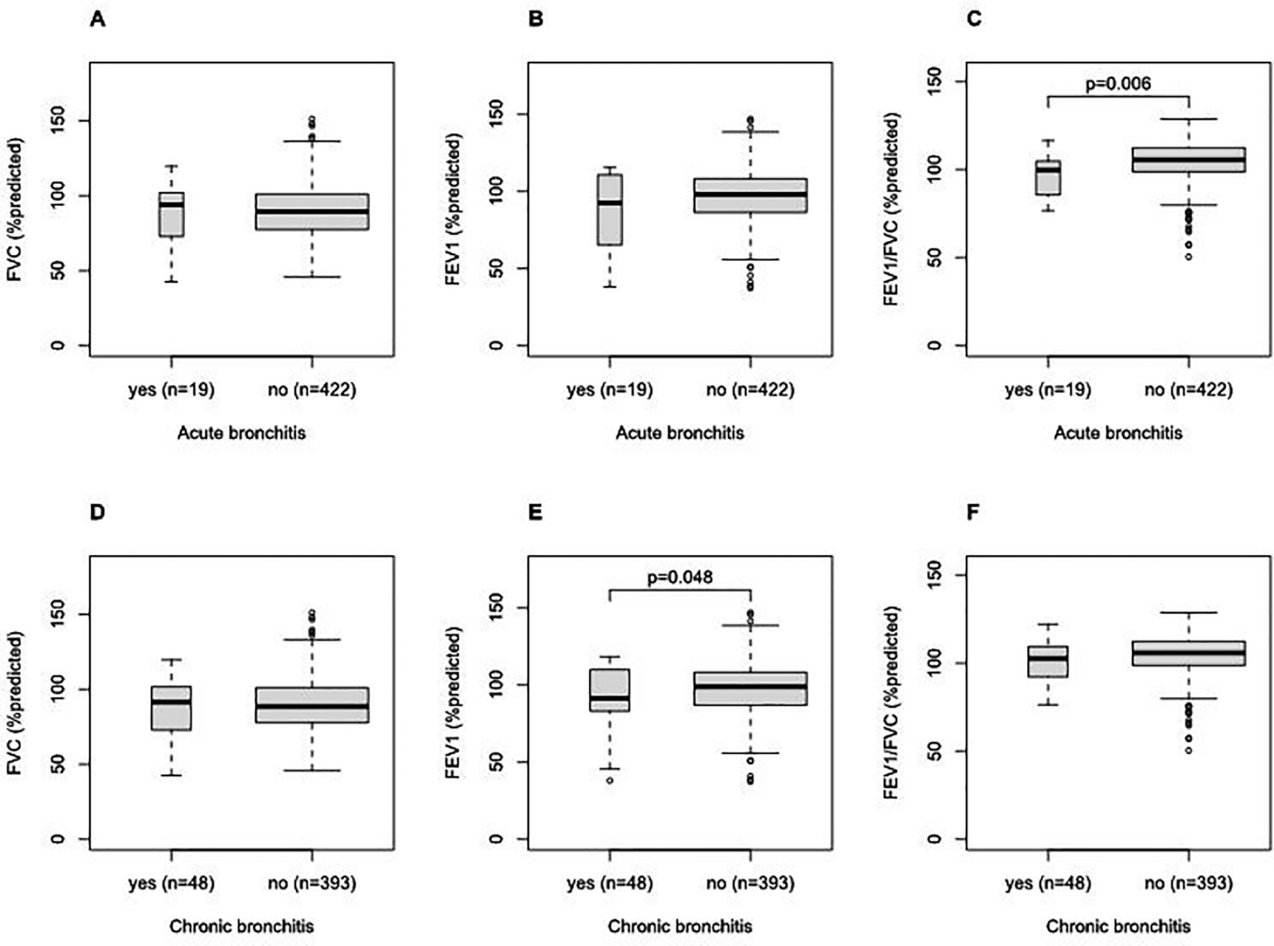
The comparison between lung function parameters of participants who answered bronchitis symptoms in their questionnaires and the same parameters of those who did not. (A) FVC and acute bronchitis; (B) FEV1 and acute bronchitis; (C) FVC/FEV1 and acute bronchitis; (D) FVC and chronic bronchitis; (E) FEV1 and chronic bronchitis; (F) FEV1/FVC and chronic bronchitis. P-values of t-tests were shown if there were significant differences between the means of the two groups.

Fig 4 compares the lung function parameters of participants who answered bronchitis symptoms in their questionnaires with the same parameters of those who did not. The mean of FEV1/FEV (%predicted) differed depending on whether participants had acute bronchitis symptoms (p = 0.006 with t-test) (Fig 4C). The mean of FEV1 (%predicted) differed depending on whether participants had chronic bronchitis symptoms (p = 0.048 with t-test) (Fig 4E). These results indicated that a decrease in FEV1/FVC (%predicted) was associated with acute bronchitis symptoms answered in questionnaires, and a decrease in FEV1 (%predicted) was associated with chronic bronchitis symptoms answered in questionnaires.

#### Lung function parameters and PM concentrations measured by personal air sampling.

**Fig 5:**
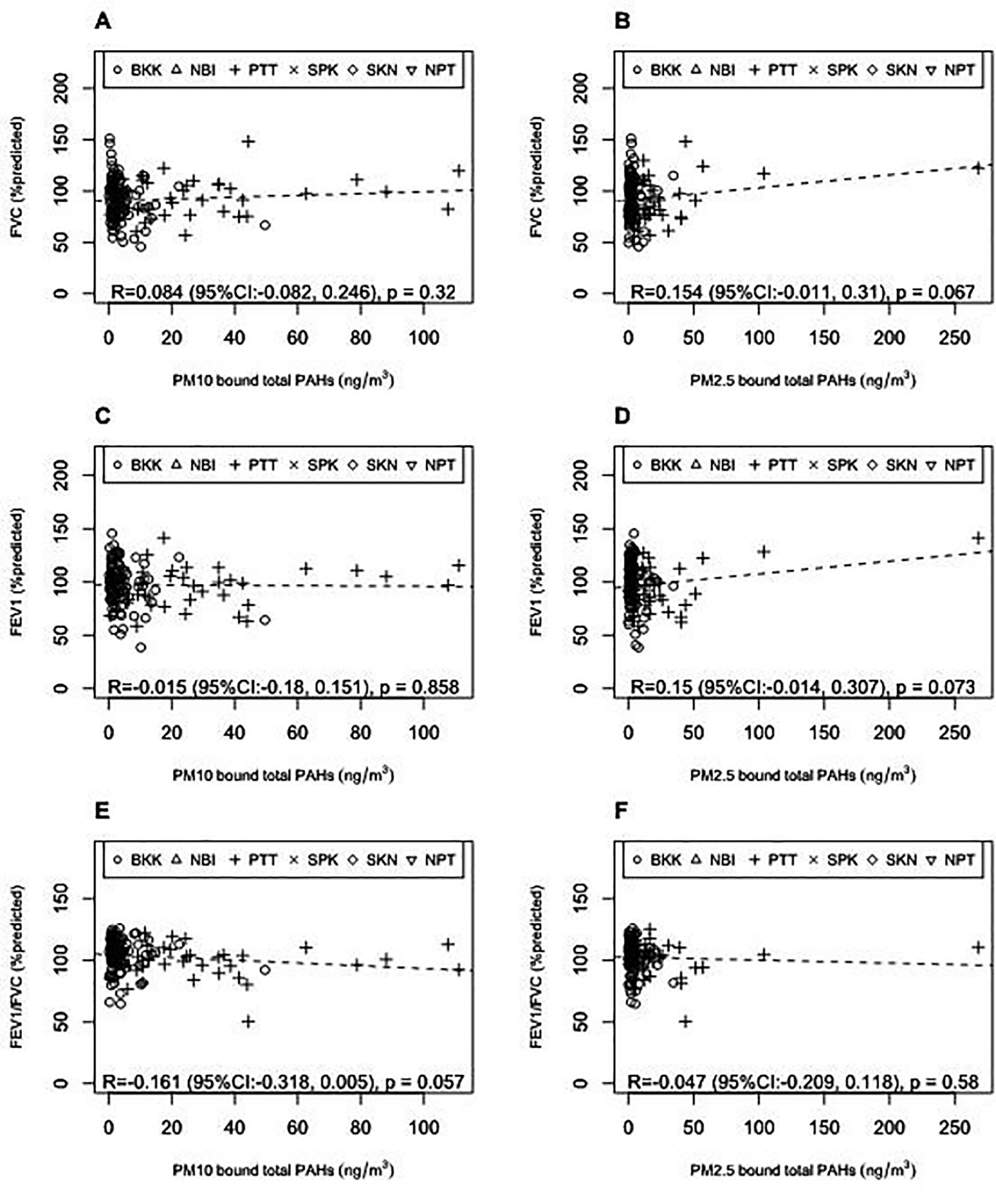
The relationship between lung function parameters (%predicted) and the exposure of PM concentrations measured by personal air sampling. (A) FVC and PM_10_-bound total PAHs; (B) FVC and PM_2.5_-bound total PAHs; (C) FEV1 and PM_10_-bound total PAHs; (D) FEV1 and PM_2.5_-bound total PAHs; (E) FEV1/FVC and PM_10_-bound total PAHs; (F) FEV1/FVC and PM_2.5_-bound total PAHs.

Fig 5 shows the relationship between the lung function parameters and the exposure of PM concentrations measured by personal air sampling. FVC, FEV1, and FEV1/FVC (%predicted) were correlated significantly with none of the PM_10_ concentration, PM_2.5_ concentration, PM_10−_bound total PAH concentration, or PM_2.5_-bound total PAH concentrations measured with personal air sampling. Personal air sampling was conducted for only one day for each person. It may take a long time for lung function parameters to be affected by the exposure to PM. A longitudinal study will be needed to measure the effect of PM exposure to the lung function parameters.

However, smoking is a well-established risk factor for impaired lung function. To account for this potential confounder, independent t-tests were conducted to assess the association between smoking status and lung function parameters. The analyses were stratified by the type of PM₁₀, PM₂.₅, PM₁₀-bound total PAHs, and PM₂.₅-bound total PAHs measured using personal air sampling. Across all subgroups, no statistically significant differences were observed in %predicted FVC, %predicted FEV₁, or %predicted FEV₁/FVC between smokers and non-smokers (S3 Fig). Several factors may explain this counterintuitive finding. First, the proportion of current smokers in our study population was relatively small, which may have limited the statistical power to detect significant differences. Second, chronic occupational exposure to traffic-related air pollutants among motorcycle taxi drivers may have had a stronger impact on lung function than smoking status, potentially masking the expected association. Third, smoking history was self-reported, which could have introduced misclassification bias. Finally, a healthy worker effect may have influenced our sample, as individuals with more severe smoking-related health problems may have already left this occupation. Therefore, while smoking remains a well-established risk factor for impaired lung function, our findings suggest that in this specific high-exposure occupational group, the effects of traffic-related air pollution may outweigh those of smoking status.

#### Association of lung function parameters to demographics and working characteristics.

S11-S13 Tables show categorical variables significantly associated with the means of percent predicted FVC, percent predicted FEV1, and percent predicted FEV1/FVC. The mean of percent predicted FVC was significantly associated with province, workstation, hairy pet, age group, hypertension, chest pain, history of allergy, and road type (p < 0.001, < 0.001, 0.041, < 0.001, < 0.001, 0.010, 0.004 and 0.012, respectively). Percent predicted FEV1 was significantly associated with age group, hypertension, history of asthma, history of allergy, neuromuscular, and chronic bronchitis (p < 0.001, 0.029, 0.018, 0.012, 0.013, and 0.048, respectively). Percent predicted FEV1/FVC was significantly associated with province, workstation, vaccine COVID-19, history of asthma, road type, incense smoke in the house, persistent phlegm, and acute bronchitis (p < 0.001, < 0.001, 0.010, 0.013, 0.002, 0.024, 0.039, and 0.006, respectively).

S14-S16 Tables show numerical variables significantly associated with a lung function parameter. Age was negatively correlated with the percent predicted FVC with Pearson’s correlation coefficient of −0.156 (95%CI: −0.246, −0.063) (p = 0.001) and the percent predicted FEV1 with −0.146 (95%CI: −0.236, −0.053) (p = 0.002). The Diastolic blood pressure was negatively correlated with the percent predicted FVC with Pearson’s correlation coefficient of −0.131 (95%CI: −0.222, −0.038) (p = 0.006) and the percent predicted FEV1 with −0.124 (95%CI: −0.215, −0.031) (p = 0.009). The work experience was negatively correlated with the percent predicted FVC with Pearson’s correlation coefficient of −0.096 (95%CI: −0.187, −0.002) (p = 0.045). The cigarette amount per day and the percent predicted FEV1/FVC with Pearson’s correlation coefficient of −0.106 (95%CI: −0.198, −0.013) (p = 0.026). The duration of smoking and the percent predicted FEV1/FVC with Pearson’s correlation coefficient of −0.112 (95%CI: −0.203, −0.019) (p = 0.018).

## Discussion

This study investigated the PM exposure of motorcycle taxi drivers in six provinces in Thailand. The data on demographics, working characteristics, and symptoms of 441 motorcycle taxi drivers were collected using questionnaires. Using personal air sampling pumps installed to the subset of the motorcycle taxi drivers, we measured the PM_10_ exposure of 151 participants and the PM_2.5_ exposure of 153 participants during their service. The associations between participants’ PM exposure and their health status, such as lung function parameters, were analyzed.

First, we found that the mean of PM_2.5_ concentrations, PM_10_-bound total PAH concentrations, and PM_2.5_-bound total PAH concentrations measured by personal air sampling differed among six provinces (Fig 1). PM concentrations measured by area air sampling were correlated with those observed at the nearest air quality monitoring station on the same days for both PM_2.5_ and PM_10_ (S1 Fig). On the other hand, the PM_10_ and PM_2.5_ concentrations measured by personal air sampling are independent of concentrations monitored at air quality monitoring stations or area air sampling devices (S2 Fig). The differences observed between personal PM measurements and those from air quality monitoring (AQM) stations can be attributed to variations in sampling context and spatial resolution. Personal samplers capture localized exposures in the breathing zone during real-world activities, particularly near high-traffic roads. In contrast, AQM stations represent broader ambient conditions and may overlook transient or street-level pollution peaks. This highlights the importance of personal exposure monitoring in occupational settings, especially for mobile workers such as motorcycle taxi drivers, whose exposures may not be accurately reflected by fixed-site measurements. This divergence is conceptually related to the “personal cloud effect” commonly reported in indoor air quality studies [51,52], where personal monitors record higher concentrations than stationary monitors due to individual activity and proximity to emission sources. Although this effect is predominantly described in indoor environments, a similar phenomenon is plausible in outdoor occupational contexts. For motorcycle taxi drivers, whose activities occur predominantly in high-traffic, street-level environments, personal exposure levels are influenced by spatial and temporal variations in emissions that fixed monitors cannot detect. For example, Samut Prakan (SPK) had the highest concentrations for PM_2.5_ and PM_10_ measured at the air quality monitoring station (S2 Fig), but it had the lowest mean of PM_10_ and PM_2.5_ concentrations in personal air sampling. The PM exposure levels of motorcycle taxi drivers depend on the PM levels on the roads where they work.

PAHs detected by personal air sampling are possibly derived not only from motorcycle’s exhaust but also from the exhaust of other vehicles on the road, such as cars, trucks, and buses, as well as ambient air pollution. Among the six provinces we studied, Pathum Thani had the highest means of total PM_10−_bound PAH concentrations (S4 Table) and total PM_2.5_-bound PAH concentrations (S5 Table). The mean of PM_10−_bound PAH concentrations in Pathum Thani was 38.4 ng/m^3^ (S4 Table), and the mean of the other five provinces was 3.78 ng/m^3^. The mean of Pathum Thani was significantly higher than Bangkok (<0.001 with t-test), Nonthaburi (p < 0.001 with t-test), Samut Prakan (p < 0.001 with t-test), Samut Sakhon (p < 0.001 with t-test), and Nakhon Pathom (p < 0.001 with t-test) (Fig 1 (C)). The mean of PM_2.5_-bound PAH concentrations in Pathum Thani was 36.9 ng/m^3^ (S5 Table), and the mean of the other five provinces was 3.02 ng/m^3^. The mean of Pathum Thani was also significantly higher than Bangkok (p = 0.009 with t-test), Nonthaburi (p = 0.003 with t-test), Samut Prakan (p = 0.004 with t-test), Samut Sakhon (p = 0.003 with t-test), and Nakhon Pathom (p = 0.003 with t-test) (Fig 1 (D)). A possible reason for the high PAH concentrations in Pathum Thani is the high percentage of heavy vehicles on the roads near the workstations of motorcycle taxi drivers in this province. According to the traffic data obtained from the Department of Highways, Thailand, the percentage of heavy vehicles at the two sampling points near workstations in Pathum Thani was 59.9%, while that of the other 115 sampling points in the six provinces we studied was 19.7% [53]. The motorcycle taxi drivers from Pathum Thani may be directly exposed to PM-bound PAH-originating diesel exhaust emissions from heavy vehicles. However, further studies are required to identify the measured sources of the pollutants. All participants from Pathum Thani wore masks during their service (S3 Table). The effect of masks on filtering PM-bound PAHs from diesel exhaust is unknown.

Previous studies found seasonal differences in the PM-bound PAH concentrations. Svecova et al. reported that the police officers in Karvina and Ostrava in the Czech Republic were exposed to PM_2.5_-bound benzo(a) pyrene at 0.6 and 0.1 ng/m^3^, respectively, in the summer season, at 6.9 and 0.8 ng/m^3^, respectively in the winter season [54]. Karageorgou et al. reported that car drivers, bus passengers, and cyclists in Thessaloniki, Greece, were exposed to PM_4_-bound benzo(a) pyrene at an average of 0.2 ng/m^3^, 0.5 ng/m^3^, and 0.4 ng/m^3^, respectively in the warm season at 1.3 ng/m^3^, 1.5 ng/m^3^, and 2.2 ng/m^3^ in the cold season [17]. Other studies showed similar trends [55–58]. Karageorgou et al. discussed that the increase in the cold season in benzo(a) pyrene concentrations was related to a higher exhaust emission rate and lower dispersion rate of chemicals at cold temperatures compared to a hot temperatures [17]. These observations suggest that PM-bound PAH concentrations must be assessed together with temperature. In our study, the PAH concentrations were measured in the winter season in Thailand, but the 24-hour average temperature at sampling sites in the studied period was 28.8°C. In the hottest months, the average temperature in Karvina and Ostrava in the Czech Republic is 18.9°C [59,60], and the average temperature in Thessaloniki in Greece is 26.1°C [61]. Given the high temperature at our sampling sites, the PM-bound PAH concentrations in Pathum Thani need to be compared with data from Karvina and Ostrava in summer and Thessaloniki in warm seasons. The investigation of PAH concentrations at our sampling sites in other seasons remains a topic for future research.

Among 15 PAH chemicals we investigated, one of the most critical chemicals is benzo(a) pyrene because it is carcinogenic to humans [33]. Among six provinces, the means of PM_10−_bound and PM_2.5_-bound benzo(a) pyrene concentrations were highest in Pathum Thani. The mean of PM_10−_bound benzo(a) pyrene in Pathum Thani was 0.581 (ng/m^3^) (S9 Table), and the mean of PM_2.5_-bound benzo(a) pyrene concentration was 1.141 (ng/m^3^) (S10 Table). Petit et al. estimated the concentration of PM-bound benzo(a) pyrene exposure for various occupations in France [62]. The motorcycle taxi drivers in Pathum Thani were exposed to a similar concentration of PM_2.5_-bound benzo(a) pyrene to heavy truck mechanic (1.3 ng/m^3^) and roadwork mechanic (0.96 ng/m^3^) in France. The ILCR of PM_2.5_-bound PAH and PM_10−_bound PAH exposure in all six provinces, including Pathum Thani, were acceptable levels (Table 5).

The PM_2.5_-bound benzo(a) pyrene equivalent (BaP_eq_) concentration in Pathum Thani was 2.0 ng/m^3^. From this concentration, the ILCR of PM_2.5_-bound PAH in Pathum Thani was estimated to be 7.8 × 10^−8^. Wang et al. reported that the BaP_eq_ concentration in Anshan City, China, in the winter season was 38.54 ng/m^3,^ and the ILCR for adults was 1.19 × 10^−5^ [63]. However, this value ILCR was calculated by assuming an exposure duration of 30 years and an exposure frequency of 365 days per year with BaP_eq_ concentration in winter. BaP_eq_ concentration in Anshan in summer was 1.4 ng/m^3^, which was lower than the BaP_eq_ concentration in Pathum Thani in this study. Zhang et al. reported that the annual BaP_eq_ of PM_2.5_-bound PAH in ambient air in Wuhan, China, was 3.48 ng/m^3^. The lifetime lung cancer risk (LLCR) in Wuhan was calculated to be 3.03 × 10^−4^. The LLCR was calculated using an inhalation unit risk (IUR_BaP_) of 8.70 × 10^−5^ ng/m^3^ [14,64]. If we use the same calculation, the LLCR of motorcycle taxi drivers in Pathum Thani is estimated to be 1.74 × 10^−4^. However, it is known that LLCR using IUR_BaP_ overestimates cancer risk [64].

Province averages of PM_10_-bound total PAH exposure of motorcycle taxi drivers during service were positively correlated with the proportions of participants who answered symptoms of chronic bronchitis in the province (Fig 3(C)). However, PM_10_-bound total PAH concentrations of participants who answered chronic bronchitis symptoms in their questionnaires were not significantly different from PM_10_-bound total PAH concentrations of those who did not (S5(G) Fig). These results indicated that an individual’s exposure to PM_10_-bound total PAH measured on a single day was not significantly associated with chronic bronchitis symptoms. Pathum Thani has significantly higher proportions of participants who answered symptoms of chronic bronchitis compared to the other five provinces (p = 0.001 by Fisher’s exact test). The motorcycle taxi drivers in Pathum Thani may have a chance to be constantly exposed to higher concentrations of PM-bound PAH than in other provinces, and the constant exposure to PM_10_-bound PAH is a possible cause of higher rates of chronic symptoms.

None of individuals’ lung function parameters, FVC (%predicted), FEV1 (%predicted), and FEV1/FVC (%predicted), was significantly correlated with the individuals’ PM_10_ concentration (S7(A)(C)(E) Fig), PM_2.5_ concentration (S7(B)(D)(F) Fig), PM_10−_bound total PAH concentration (Fig 5(A)(C)(E)), or PM_2.5_-bound total PAH concentrations (Fig 5(B)(D)(F)). Personal air sampling was conducted on a single day for each person. It may take a long time for lung function parameters to be affected by exposure to PM. The FVC (%predicted) of participants were significantly correlated with their work experience (year) with Pearson’s correlation of −0.096 (95%CI: −0.187, −0.002) (p = 0.045). A longitudinal study will be needed to measure the effect of PM exposure on lung function parameters more precisely.

Acute and chronic bronchitis symptoms were associated significantly with some lung function parameters. Symptoms of chronic bronchitis were significantly associated with the percent predicted FEV1 (p = 0.048) (Fig 4(E)). This association is consistent with previous studies [65,66]. Symptoms of acute bronchitis were significantly associated with the percent predicted FEV1/FVC (p = 0.006) (Fig 4(C)). It is known that FEV1 decreased from acute bronchitis [65–70]. However, symptoms of acute bronchitis were not significantly associated with the percent predicted FEV1 in this study (Fig 4(B)).

Our study showed that province means of PM_10_-bound PAH concentrations were significantly associated with percentages of chronic bronchitis symptoms in the provinces (Fig 3), but province means of any PM-bound PAH concentrations were significantly correlated with none of the province means of lung function parameters (S8 Fig). Sunyer et al. also reported that exposure to dust, gases, and fumes dust was associated with the incidence of chronic bronchitis without decreasing lung function parameters using data from more than 8,000 participants in Europe [71]. The causal relationship between motorcycle taxi drivers’ PM and PM-bound PAH exposure in Pathum Thani and their respiratory symptoms needs to be further investigated.

In our previous study, we reported that motorcycle taxi drivers in Bangkok had the highest mean respirable dust concentration among six central provinces in Thailand during the rainy season [25]. Bangkok had the highest hazard quotient value for a non-carcinogenic risk to human health caused by respirable dust exposure among the six provinces in the rainy season [26]. On the other hand, this study found that Pathum Thani had the highest mean of PM_10_ concentration, PM_2.5_ concentrations, PM_10_-bound total PAHs, and PM_2.5_-bound total PAHs among six provinces in the winter season. Pathum Thani had the highest ILCR of PM_10_-bound total PAHs and PM_2.5_-bound total PAHs among six provinces. We cannot directly compare the result of this study and our previous studies because the target of PM and the sampling seasons are different. However, Pathum Thani had the lowest percent predicted FEV1 among six provinces in both the rainy season [25] and the winter season (this study, Table 6). Seasonal differences in the concentration of PM and PM-bound PAH should be analyzed in a coherent manner in the future.

This study has the following limitations. First, this study conducted personal air sampling on a single day for each person. However, the PM exposure of each person may differ from day to day. The province average of PM exposure may also change depending on the measurement day. In order to solve this problem, personal air sampling should be repeated several times. Longitudinal studies are needed to follow up on the lung function parameters of participants more than once to clarify the association between PM exposure and lung function parameters. Second, due to equipment limitations, not all participants were involved in personal air sampling. Some associations may not be significant because of the small sample numbers. Third, our study used a questionnaire to investigate acute and chronic bronchitis symptoms. Diagnosis by a medical doctor will be needed for more accurate evaluation of the association between PM-bound PAH exposure and chronic or acute bronchitis. Lastly, we only estimated the inhalation of incremental lifetime cancer risk of benzo(a) pyrene. Benzo(a) pyrene can enter the human body via ingestion and dermal, so our result may underestimate the overall cancer risk.

## Conclusions

This study recruited motorcycle taxi drivers in Thailand and conducted personal air sampling to measure the PM and PM-bound PAH exposures. We found that the PM_10_ and PM_2.5_ concentrations measured by personal air sampling were independent of PM_10_ and PM_2.5_ concentrations monitored at air quality monitoring stations or those measured by area air sampling devices. Pathum Thani had significantly higher means of total PM_10−_bound PAH concentrations and total PM_2.5_-bound PAH concentrations than the other five provinces. The incremental lifetime cancer risk due to PM_2.5_-bound PAH and PM_10−_bound PAH exposure in Pathum Thani was 4.5 × 10^−8^ and 7.8 × 10^−8^, respectively, which were the acceptable risk level (<1.0 × 10^−6^). PM-bound PAH exposure of motorcycle taxi drivers in Pathum Thani must be reduced. Province means of PM-bound PAH concentrations were significantly correlated with none of the province means lung function parameters. However, province means of PM_10_-bound PAH concentrations were significantly associated with percentages of chronic bronchitis symptoms in the provinces. The lung function parameters of motorcycle taxi drivers in Pathum Thani must be followed up on in the future. At the individual level, none of the individual lung function parameters showed a significant association with PM or PM-bound PAH exposure concentrations. This may be due to limitations of the cross-sectional design, which captures only short-term exposure through one-day personal air sampling. Since respiratory effects from PM exposure often develop over prolonged periods, a single-day measurement may not accurately represent cumulative exposure. Further longitudinal studies with repeated sampling are needed to clarify these relationships.

## Supporting information

S1 Fig The relationship between daily PM concentrations measured by AAS and AQM. (TIFF)

S2 Fig The relationship between mean PM10 and PM2.5 concentrations measured by PAS, AAS, and AQM. (TIFF)

S3 Fig The comparison between the lung function parameters of participants who smoke and those of participants who did not. (TIFF)

S4 Fig The relationship between PM10-bound PAHs measured by personal air sampling from six provinces. (TIFF)

S5 Fig The comparison of PM concentrations measured by personal air sampling between participants who answered bronchitis symptoms in their questionnaires and those who did not. (TIFF)

S6 Fig The relationship between the percentage of participants who answered symptoms of chronic bronchitis in their questionnaires and the mean of PM concentrations measured by personal air sampling in each province. (TIFF)

S7 Fig The relationship between lung function parameters (%predicted) and the exposure of PM concentrations measured by personal air sampling. (TIFF)

S8 Fig The relationship between lung function parameters (%predicted) and the exposure of PM-bound total PAHs concentrations measured by personal air sampling. (TIFF)

S9 Fig Locations of personal air sampling workstations across six provinces. (TIF)

S10 Fig Workstation of motorcycle taxi drivers. (TIF)

S11 Fig Motorcycle taxi driver wearing a personal air sampler during service: (a) PM₁₀, (b) PM_2.5_. (TIFF)

S1 Table Summary of previous studies investigating PM exposure and incremental lifetime cancer risk (ILCR). (DOCX)

S2 Table Number of personal air samples collected at each workstation. (DOCX)

S3 Table Demographics and working characteristics of participants (n = 441). (DOCX)

S4 Table The concentration of PM_10_ and PM_10_-bound total PAHs from personal air sampling. (DOCX)

S5 Table The concentration of PM_2.5_ and PM_2.5_-bound total PAHs from personal air sampling. (DOCX)

S6 Table Total BaPeg for PM_10_-bound PAH in six provinces. (DOCX)

S7 Table Total BaPeg for PM_2.5_-bound PAH in six provinces. (DOCX)

S8 Table Parameters used for calculating LADD and ILCR. (DOCX)

S9 Table The concentration of PM_10_-bound PAHs (ng/m^3^). (DOCX)

S10 Table The concentration of PM_2.5_-bound PAHs (ng/m^3^). (DOCX)

S11 Table Association between categorical variables and FVC (%predicted). (DOCX)

S12 Table Association between categorical variables and FEV1 (%predicted). (DOCX)

S13 Table Association between categorical variables and FEV1/FVC (%predicted). (DOCX)

S14 Table Association between numerical variables and FVC (%predicted). (DOCX)

S15 Table Association between numerical variables and FEV1 (%predicted). (DOCX)

S16 Table Association between numerical variables and FEV1/FVC (%predicted). (DOCX)
