# Supplementary figures and images for: Analysis of PM-bound polycyclic aromatic hydrocarbons exposure among motorcycle taxi drivers in six central provinces in Thailand in winter
Source: PLoS One. 2025 Dec 1;20(12):e0336587. doi: 10.1371/journal.pone.0336587 (PMC12668520; doi:10.1371/journal.pone.0336587)

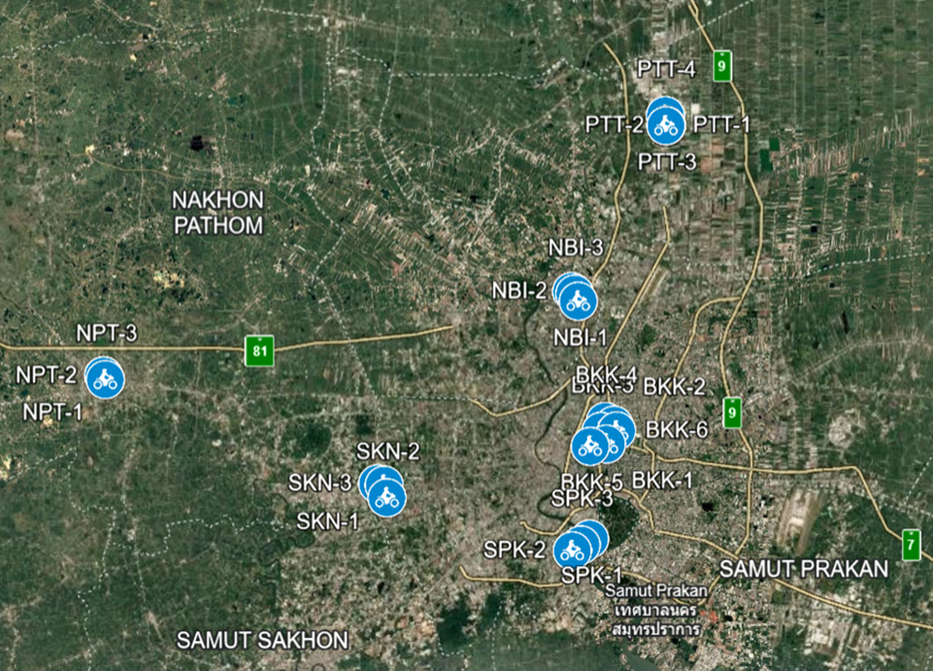

Supplement: S9 Fig — (TIF) [file pone.0336587.s009.tif]

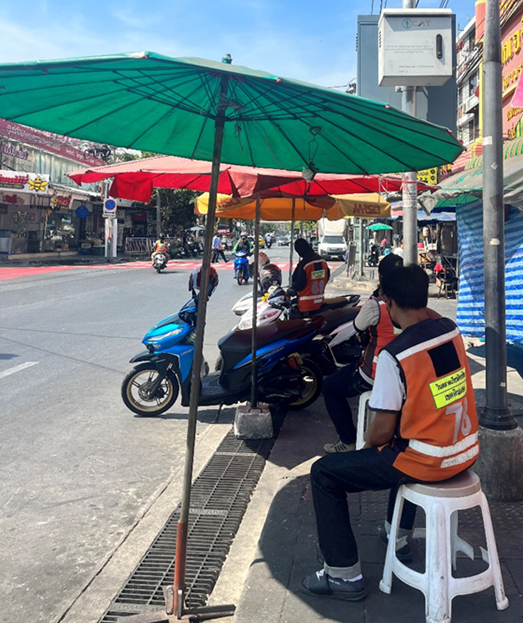

Supplement: S10 Fig — (TIF) [file pone.0336587.s010.tif]

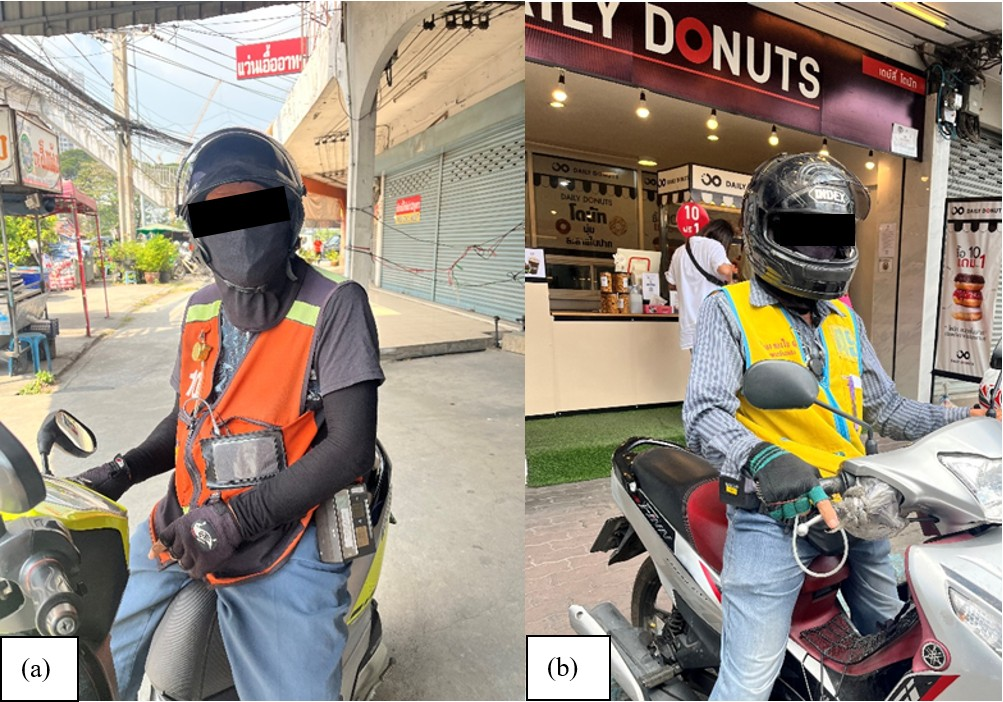

Supplement: S11 Fig — (TIFF) [file pone.0336587.s011.tiff]
