# Supplementary material for: Analysis of PM-bound polycyclic aromatic hydrocarbons exposure among motorcycle taxi drivers in six central provinces in Thailand in winter
Source: PLoS One. 2025 Dec 1;20(12):e0336587. doi: 10.1371/journal.pone.0336587 (PMC12668520; doi:10.1371/journal.pone.0336587)
Supplement: S1 Table — (DOCX) [file pone.0336587.s012.docx]

**S1 Table.** **Summary of previous studies investigating PM exposure and incremental lifetime cancer risk (ILCR)**

| Authors (year) | Country | Sampling method | Summary of findings |
| --- | --- | --- | --- |
| Chuersuwan et al. (2008) | Thailand | Area air sampling | Automobile emissions and biomass burning each contributed approximately 33% to the PM_10_ concentrations. Similarly, automobile emissions accounted for 32% and biomass burning for 26% of the PM_2.5_ concentrations [1]. |
| Duangkaew  (2012) | Thailand | Area air sampling | During the wet season, the average ratio of organic carbon to elemental carbon (OC/EC) in PM_10_ and PM_2.5_ was 1.77 and 1.56, respectively. The major fractions of the eight elemental carbon components were EC1 and EC2, which are primarily associated with motor vehicle exhaust emissions [2]. |
| Suwanthanee (2002) | Thailand | Area air sampling | Chemical mass balance receptor modeling indicated that diesel and motorcycle emissions were the predominant sources during the northeast and southwest monsoon seasons, contributing 17-41% (22-114 μg/m³) and 18-35% (14-48 μg/m³) of particulate matter concentrations, respectively [3]. |
| Thongyen  (2009) | Thailand | Area air sampling | Traffic emissions from four-stroke motorcycles, tuk-tuks, compressed natural gas vehicles, and two-stroke motorcycles were identified as primary sources of polycyclic aromatic hydrocarbons (PAHs) in Bangkok [4] |
| Wang et al.  (2023) | China | Area air sampling | The incremental lifetime cancer risk (ILCR) associated with PM₂.₅-bound polycyclic aromatic hydrocarbons (PAHs) in urban residential areas of China was estimated at 7.45 × 10⁻⁵ [5]. |
| Taghvaee et al. (2018) | Tehran | Area air sampling | The incremental lifetime cancer risk (ILCR) associated with PM₂.₅-bound PAHs in central Tehran was estimated to be 2.8 × 10⁻⁵ [6]. |
| Zhang et al.  (2019) | China | Area air sampling | In China, the incremental lifetime cancer risk (ILCR) associated with PM₂.₅-bound PAHs was estimated at 3.03 × 10⁻⁴, with vehicle emissions accounting for approximately 57.1% of the total risk (1.6 × 10⁻⁴ on average) [7]. |
| Ali-Taleshi et al. (2021) | Iran | Area air sampling | In Iran, the incremental lifetime cancer risk (ILCR) associated with PM₂.₅-bound PAHs ranged from 1.33 × 10⁻⁵ to 2.28 × 10⁻⁵, with the highest cancer risk attributed to emissions from heavy-duty vehicles and natural gas–coal/biomass combustion compared to other identified sources [8]. |
| Onaiwu and Eferavware  (2023) | Nigeria | Area air sampling | In Benin City, Nigeria, the incremental lifetime cancer risk (ILCR) of PM₂.₅-bound PAHs among adults, based on area air sampling in automobile workshops, was estimated at 3.58 × 10⁻⁵ during the dry season and 2.80 × 10⁻⁵ during the wet season [9]. |
| Karageorgou et al. (2021) | Greece | Personal air sampling | In a study conducted in Greece, winter commuting by bicycle resulted in the highest exposure to PM₄, with a mean concentration of 99.8 µg/m³, followed by bus commuting at 91.0 µg/m³ and car commuting at 37.2 µg/m³[10]. |
| Chuang et al. (2020) | Taiwan | Personal air sampling | In Taiwan, travel by scooter resulted in the highest exposure to PM₂.₅ (53.6 µg/m³), followed by walking (42.7 µg/m³), bus (34.7 µg/m³), car (26.2 µg/m³), and subway (21.7 µg/m³) [11]. |
| Kinney et al. (2011) | Kenya | Personal air sampling | In Kenya, walking at street level resulted in substantially higher PM₂.₅ exposure (119.5 µg/m³) compared to walking on a third-floor rooftop (42.8 µg/m³) [12]. |
| Wu et al.  (2021) | Taipei | Personal air sampling | Commuting motorcyclists in Taipei experienced higher PM₂.₅ exposure (27.65 µg/m³) compared to cyclists (23.27 µg/m³) [13]. |

**References**

1. Chuersuwan N, Nimrat S, Lekphet S, Kerdkumrai T. Levels and major sources of PM2.5 and PM10 in Bangkok Metropolitan Region. Environment International. 2008;34:671–7.

2. Duangkaew S. Carbon compositions in the Bangkok urban background ambient air at Chulalongkorn University: Chulalongkorn University; 2012.

3. Suwanthanee P. Total suspended particulate matter source apportionment in Bangkok metropolitan area: Chulalongkorn University; 2002.

4. Thongyen T. Source apportionment of Polycyclic Aromatic Hydrocarbons in size-selected particulate matter in Bangkok metropolitan area: Chulalongkorn University; 2009.

5. Wang T, Zhang L, Zhang P, Yu G, Chen C, Qin X, et al. Unveiling the pollution and risk of atmospheric (gaseous and particulate) polycyclic aromatic hydrocarbons (PAHs) in a heavily polluted Chinese city: A multi-site observation research. Journal of Cleaner Production. 2023;428:139454.

6. Taghvaee S, Sowlat MH, Hassanvand MS, Yunesian M, Naddafi K, Sioutas C. Source-specific lung cancer risk assessment of ambient PM2.5-bound polycyclic aromatic hydrocarbons (PAHs) in central Tehran. Environment International. 2018;120:321-32.

7. Zhang Y, Zheng H, Zhang L, Zhang Z, Xing X, Qi S. Fine particle-bound polycyclic aromatic hydrocarbons (PAHs) at an urban site of Wuhan, central China: Characteristics, potential sources and cancer risks apportionment. Environmental Pollution. 2019;246:319-27.

8. Ali-Taleshi MS, Riyahi Bakhtiari A, Moeinaddini M, Squizzato S, Feiznia S, Cesari D. Single-site source apportionment modelling of PM2.5-bound PAHs in the Tehran metropolitan area, Iran: Implications for source-specific multi-pathway cancer risk assessment. Urban Climate. 2021;39:100928.

9. Onaiwu GE, Eferavware SA. The potential health risk assessment of PM2.5-bound polycyclic aromatic hydrocarbons (PAHs) on the human respiratory system within the ambient air of automobile workshops in Benin City, Nigeria. Air Quality, Atmosphere & Health. 2023;16(12):2431-41.

10. Karageorgou K, Manoli E, Kouras A, Samara C. Commuter exposure to particle-bound polycyclic aromatic hydrocarbons in Thessaloniki, Greece. Environmental Science and Pollution Research. 2021;28(42):59119-30.

11. Chuang K-J, Lin L-Y, Ho K-F, Su C-T. Traffic-related PM2.5 exposure and its cardiovascular effects among healthy commuters in Taipei, Taiwan. Atmospheric Environment: X. 2020;7:100084.

12. Kinney PL, Gichuru MG, Volavka-Close N, Ngo N, Ndiba PK, Law A, et al. Traffic impacts on PM2.5 air quality in Nairobi, Kenya. Environmental Science & Policy. 2011;4:369-78.

13. Wu T-G, Chang J-C, Huang S-H, Lin W-Y, Chan C-C, Wu C-F. Exposures and health impact for bicycle and electric scooter commuters in Taipei. Transportation Research Part D. 2021;91:102696.
