# Supplementary material for: Analysis of PM-bound polycyclic aromatic hydrocarbons exposure among motorcycle taxi drivers in six central provinces in Thailand in winter
Source: PLoS One. 2025 Dec 1;20(12):e0336587. doi: 10.1371/journal.pone.0336587 (PMC12668520; doi:10.1371/journal.pone.0336587)
Supplement: S2 Table — (DOCX) [file pone.0336587.s013.docx]

**S2 Table.** **Number of personal air samples collected at each workstation.**

| Province | Workstation | Number of personal air samples collected at each workstation (sample) | |
| --- | --- | --- | --- |
|  |  | PM_10_ | PM_2.5_ |
| Bangkok | BKK-1 | 10 | 10 |
|  | BKK-2 | 17 | 20 |
|  | BKK-3 | 7 | 7 |
|  | BKK-4 | 7 | 7 |
|  | BKK-5 | 2 | 2 |
|  | BKK-6 | 4 | 4 |
| Nonthaburi | NBI-1 | 7 | 7 |
|  | NBI-2 | 4 | 4 |
|  | NBI-3 | 12 | 7 |
| Pathum Thani | PTT-1 | 9 | 9 |
|  | PTT-2 | 5 | 5 |
|  | PTT-3 | 4 | 7 |
|  | PTT-4 | 4 | 4 |
| Samut Prakan | SPK-1 | 7 | 9 |
|  | SPK-2 | 16 | 14 |
|  | SPK-3 | 4 | 4 |
| Samut Sakhon | SKN-1 | 8 | 8 |
|  | SKN-2 | 1 | 1 |
|  | SKN-3 | 7 | 7 |
| Nakhon Prathom | NPT-1 | 7 | 8 |
|  | NPT-2 | 5 | 5 |
|  | NPT-3 | 4 | 4 |
|  | Total | 151 | 153 |
