# Supplementary material for: Analysis of PM-bound polycyclic aromatic hydrocarbons exposure among motorcycle taxi drivers in six central provinces in Thailand in winter
Source: PLoS One. 2025 Dec 1;20(12):e0336587. doi: 10.1371/journal.pone.0336587 (PMC12668520; doi:10.1371/journal.pone.0336587)
Supplement: S3 Table — (DOCX) [file pone.0336587.s014.docx]

**S3 Table.** **Demographics and working characteristics of participants (n=441).**

| **Characteristics** | **BKK*** | **NBI*** | **PTT*** | **SPK*** | **SKN*** | **NPT*** | **Total (%)** |
| --- | --- | --- | --- | --- | --- | --- | --- |
| Gender |  |  |  |  |  |  |  |
| Male | 216 (90.0) | 29 (85.3) | 39 (100.0) | 49 (89.1) | 29 (100.0) | 36 (81.8) | 398 (90.2) |
| Female | 24 (10.0) | 5 (14.7) | 0 (0.0) | 6 (10.9) | 0 (0.0) | 8 (18.2) | 43 (9.8) |
| Age |  |  |  |  |  |  |  |
| 20-39 | 69 (28.8) | 6 (17.6) | 5 (12.8) | 12 (21.8) | 6 (20.7) | 5 (11.4) | 103 (23.4) |
| 40-59 | 148 (61.7) | 20 (58.5) | 26 (66.7) | 28 (50.9) | 16 (55.2) | 27 (61.4) | 265 (60.1) |
| ≥60 | 23 (9.6) | 8 (23.5) | 8 (20.5) | 15 (27.3) | 7 (24.1) | 12 (27.3) | 73 (16.5) |
| Drinking alcohol |  |  |  |  |  |  |  |
| Non-drink | 47 (19.6) | 6 (17.6) | 5 (12.8) | 15 (27.5) | 7 (24.1) | 15 (34.1) | 95 (21.5) |
| Current drink | 152 (63.3) | 17 (50.0) | 25 (64.1) | 33 (60.0) | 11 (37.9) | 17 (38.6) | 255 (57.8) |
| Former drink | 41 (17.1) | 11 (32.4) | 9 (23.1) | 7 (12.7) | 11 (37.9) | 12 (27.3) | 91 (20.7) |
| Smoking history |  |  |  |  |  |  |  |
| Non-smoker | 72 (30.0) | 7 (20.6) | 8 (20.5) | 19 (34.5) | 9 (31.0) | 17 (37.7) | 132 (29.9) |
| Current smoker | 113 (47.1) | 15 (44.1) | 18 (46.2) | 23 (41.9) | 7 (24.1) | 19 (24.1) | 195 (44.2) |
| Former smoker | 55 (22.9) | 12 (35.3) | 13 (33.3) | 13 (23.6) | 13 (44.9) | 8 (18.2) | 114 (25.9) |
| History of the COVID-19 |  |  |  |  |  |  |  |
| Yes | 121 (50.4) | 11 (32.4) | 21 (53.8) | 23 (41.8) | 14 (48.3) | 18 (40.9) | 208 (47.2) |
| No | 119 (49.6) | 23 (67.6) | 18 (46.2) | 32 (58.2) | 15 (51.7) | 26 (59.1) | 233 (52.8) |
| Vaccine for COVID-19 |  |  |  |  |  |  |  |
| Yes | 203 (84.6) | 31 (91.2) | 35 (89.7) | 54 (98.2) | 27 (93.1) | 41 (93.2) | 391 (88.7) |
| No | 37 (15.4) | 3 (8.8) | 4 (10.3) | 1 (1.8) | 2 (6.9) | 3 (6.8) | 50 (11.3) |
| Physical activity |  |  |  |  |  |  |  |
| Yes | 70 (29.2) | 15 (44.1) | 18 (46.2) | 19 (34.5) | 14 (48.3) | 14 (31.8) | 150 (34.0) |
| No | 170 (70.8) | 19 (55.9) | 21 (53.8) | 36 (65.5) | 15 (51.7) | 30 (68.2) | 291 (66.0) |
| Body Mass Index; BMI |  |  |  |  |  |  |  |
| Underweight (<18.5kg/m^2^) | 14 (5.8) | 2 (5.9) | 0 (0.0) | 3 (5.5) | 1 (3.4) | 1 (2.3) | 21 (4.8) |
| Normal (18.5-24.9 kg/m^2^) | 115 (47.9) | 15 (44.1) | 25 (64.1) | 22 (40.0) | 17 (58.6) | 17 (38.6) | 211 (47.8) |
| Overweight (25.0-29.9kg/m^2^) | 66 (27.5) | 7 (20.6) | 10 (25.6) | 17 (30.9) | 9 (31.0) | 20 (45.5) | 129 (29.3) |
| Obese (>30.0kg/m^2^) | 45 (18.8) | 10 (29.4) | 4 (10.3) | 13 (23.6) | 2 (6.9) | 6 (13.6) | 80 (18.1) |
| Home near road |  |  |  |  |  |  |  |
| Main road | 193 (80.4) | 12 (35.3) | 13 (33.3) | 33 (60.0) | 9 (31.0) | 4 (9.1) | 264 (59.9) |
| Minor road | 47 (19.6) | 22 (64.7) | 26 (66.7) | 22 (40.0) | 20 (69.0) | 40 (90.9) | 177 (40.1) |
| Mosquito repellent coil |  |  |  |  |  |  |  |
| Yes | 33 (13.8) | 8 (23.5) | 16 (41.0) | 12 (21.8) | 11 (37.9) | 14 (31.8) | 94 (21.3) |
| No | 207 (86.2) | 26 (76.5) | 23 (59.0) | 43 (78.2) | 18 (62.1) | 30 (68.2) | 347 (78.7) |
| Insect repellent spray |  |  |  |  |  |  |  |
| Yes | 51 (21.2) | 10 (29.4) | 15 (38.5) | 14 (25.5) | 11 (37.9) | 21 (47.7) | 122 (27.7) |
| No | 189 (78.8) | 24 (70.6) | 24 (61.5) | 41 (74.5) | 18 (62.1) | 23 (52.3) | 319 (72.3) |
| Incense smoke in the house |  |  |  |  |  |  |  |
| Yes | 36 (15.0) | 3 (8.8) | 11 (28.2) | 20 (36.4) | 11 (37.9) | 23 (52.3) | 104 (23.6) |
| No | 204 (85.0) | 31 (91.2) | 28 (71.8) | 35 (63.6) | 18 (62.1) | 21 (47.7) | 337 (76.4) |
| Cooking with firewood |  |  |  |  |  |  |  |
| Yes | 17 (7.1) | 2 (5.9) | 4 (10.3) | 1 (1.8) | 2 (6.9) | 8 (18.2) | 34 (7.7) |
| No | 223 (92.9) | 32 (94.1) | 35 (89.7) | 54 (98.2) | 27 (93.1) | 36 (81.8) | 407 (92.3) |
| Garbage disposal |  |  |  |  |  |  |  |
| Trash | 240 (100.0) | 34 (100.0) | 38 (97.4) | 55 (100.0) | 28 (96.6) | 43 (97.7) | 438 (99.3) |
| Burning | 0 (0.0) | 0 (0.0) | 1 (2.6) | 0 (0.0) | 1 (3.4) | 1 (2.3) | 3 (0.7) |
| Hairy pet |  |  |  |  |  |  |  |
| No | 166 (69.2) | 21 (61.8) | 23 (59.0) | 29 (52.7) | 20 (69.0) | 20 (45.4) | 279 (63.3) |
| Yes, outdoor | 42 (17.5) | 4 (11.8) | 9 (23.1) | 11 (20.0) | 9 (31.0) | 11 (25.0) | 86 (19.5) |
| Yes, indoor | 32 (13.3) | 9 (26.5) | 7 (17.9) | 15 (27.3) | 0 (0.0) | 13 (29.5) | 76 (17.2) |
| Work experience |  |  |  |  |  |  |  |
| <5 years | 71 (29.6) | 10 (29.4) | 11 (28.2) | 13 (23.6) | 13 (44.8) | 9 (20.5) | 127 (28.8) |
| 5-10 years | 54 (22.5) | 7 (20.6) | 10 (25.6) | 11 (20.0) | 6 (20.7) | 7 (15.9) | 95 (21.5) |
| >10 years | 115 (47.9) | 17 (50.0) | 18 (46.2) | 31 (56.4) | 10 (34.5) | 28 (63.6) | 219 (49.7) |
| Work hour |  |  |  |  |  |  |  |
| <8 hours per day | 17 (7.1) | 2 (5.9) | 5 (12.8) | 5 (9.1) | 0 (0.0) | 5 (11.4) | 34 (7.7) |
| >8 hours per day | 223 (92.9) | 32 (94.1) | 34 (87.2) | 50 (90.9) | 29 (100.0) | 39 (88.6) | 407 (92.3) |
| Continuous drive |  |  |  |  |  |  |  |
| No break | 121 (50.4) | 12 (35.3) | 22 (56.4) | 9 (16.4) | 7 (24.1) | 25 (56.8) | 196 (44.4) |
| With breaks | 119 (49.6) | 22 (64.7) | 17 (43.6) | 46 (83.6) | 22 (75.9) | 19 (43.2) | 245 (55.6) |
| Driving type |  |  |  |  |  |  |  |
| Passenger | 116 (48.3) | 16 (47.1) | 22 (56.4) | 38 (69.1) | 14 (48.3) | 18 (40.9) | 224 (50.8) |
| Passenger and parcel | 124 (51.7) | 18 (52.9) | 17 (43.6) | 17 (30.9) | 15 (51.7) | 26 (59.1) | 217 (49.2) |
| Parttime job |  |  |  |  |  |  |  |
| Yes | 58 (24.2) | 6 (17.6) | 10 (25.6) | 11 (20.0) | 6 (20.7) | 9 (20.5) | 100 (22.7) |
| no | 182 (75.8) | 28 (82.4) | 29 (74.4) | 44 (80.0) | 23 (79.3) | 35 (79.5) | 341 (77.3) |
| Type of mask |  |  |  |  |  |  |  |
| N95 mask | 0 (0.0) | 0 (0.0) | 2 (5.1) | 0 (0.0) | 0 (0.0) | 0 (0.0) | 2 (0.5) |
| Double | 50 (20.8) | 1 (2.9) | 3 (7.7) | 7 (12.7) | 12 (41.4) | 0 (0.0) | 54 (12.2) |
| Surgical mask | 161 (67.1) | 28 (82.4) | 28 (71.8) | 42 (76.4) | 14 (48.3) | 38 (86.4) | 311 (70.5) |
| Cotton mask | 28 (11.7) | 5 (14.7) | 6 (15.4) | 6 (10.9) | 3 (10.3) | 6 (13.6) | 54 (12.2) |
| No mask | 1 (0.4) | 0 (0.0) | 0 (0.0) | 0 (0.0) | 0 (0.0) | 0 (0.0) | 1 (0.2) |
| Type of helmet |  |  |  |  |  |  |  |
| Full face | 23 (9.6) | 5 (14.7) | 7 (17.9) | 11 (20.0) | 8 (27.6) | 6 (13.6) | 60 (13.6) |
| Open face | 101 (42.1) | 21 (61.8) | 24 (61.5) | 32 (58.2) | 16 (55.2) | 27 (61.4) | 221 (50.1) |
| Half face | 116 (48.3) | 8 (23.5) | 8 (20.5) | 12 (21.8) | 5 (17.2) | 11 (25.0) | 160 (36.3) |

*Bangkok (BKK), Nonthaburi (NBI), Pathum Thani (PTT), Samut Prakan (SPK), Samut Sakhon (SKN), and Nakhon Pathom (NPT)
