# Supplementary material for: Analysis of PM-bound polycyclic aromatic hydrocarbons exposure among motorcycle taxi drivers in six central provinces in Thailand in winter
Source: PLoS One. 2025 Dec 1;20(12):e0336587. doi: 10.1371/journal.pone.0336587 (PMC12668520; doi:10.1371/journal.pone.0336587)
Supplement: S4 Table — (DOCX) [file pone.0336587.s015.docx]

**S4 Table.** **The concentration of PM_10_ and PM_10_-bound total PAHs from** **personal air sampling.**

| **Province** | **n** | **PM_10_ concentration (µg/m^3^)** | | | | | **n** | **PM_10−_bound total PAHs (ng/m^3^)** | | | | |
| --- | --- | --- | --- | --- | --- | --- | --- | --- | --- | --- | --- | --- |
|  |  | **Median** | **Mean** | **SD** | **Min.** | **Max.** |  | **Median** | **Mean** | **SD** | **Min.** | **Max.** |
| Bangkok | 47 | 144.7 | 161.7 | 88.8 | 77.4 | 645.7 | 47 | 6.4 | 11.1 | 14.4 | 0.5 | 78.8 |
| Nonthaburi | 23 | 146.3 | 162.6 | 54.6 | 67.6 | 277.6 | 17 | 1.5 | 1.7 | 1.0 | 0.5 | 4.0 |
| Pathum Thani | 22 | 189.1 | 224.9 | 109.7 | 126.2 | 602.8 | 22 | 28.3 | 38.4 | 29.2 | 6.0 | 111.1 |
| Samut Prakan | 27 | 129.5 | 161.3 | 109.4 | 68.8 | 577.3 | 25 | 2.7 | 2.9 | 2.1 | 0.1 | 9.4 |
| Samut Sakhon | 16 | 146.6 | 165.8 | 58.3 | 97 | 316.6 | 14 | 1.3 | 1.6 | 1.1 | 0.1 | 4.0 |
| Nakhon Prathom | 16 | 121.8 | 169.6 | 154.1 | 81.7 | 626 | 16 | 1.2 | 1.6 | 1.2 | 0.3 | 5.0 |
| Total | 151 | 146.1 | 172.3 | 96.6 | 67.6 | 645.7 | 141 | 3.4 | 10.7 | 18.9 | 0.1 | 111.1 |
