# Supplementary material for: Analysis of PM-bound polycyclic aromatic hydrocarbons exposure among motorcycle taxi drivers in six central provinces in Thailand in winter
Source: PLoS One. 2025 Dec 1;20(12):e0336587. doi: 10.1371/journal.pone.0336587 (PMC12668520; doi:10.1371/journal.pone.0336587)
Supplement: S5 Table — (DOCX) [file pone.0336587.s016.docx]

**S5 Table.** **The concentration of PM_2.5_ and PM_2.5_-bound total PAHs from personal air sampling.**

| **Province** | **n** | **PM_2.5_ concentration (µg/m^3^)** | | | | | **n** | **PM_2.5_ bound total PAHs (ng/m^3^)** | | | | |
| --- | --- | --- | --- | --- | --- | --- | --- | --- | --- | --- | --- | --- |
|  |  | **Median** | **Mean** | **SD** | **Min.** | **Max.** |  | **Median** | **Mean** | **SD** | **Min.** | **Max.** |
| Bangkok | 50 | 125.1 | 132.2 | 54.9 | 63.2 | 340.4 | 47 | 4.6 | 6.7 | 7.1 | 0.0 | 34.5 |
| Nonthaburi | 18 | 144.1 | 206.2 | 232.9 | 94.1 | 1117.7 | 14 | 0.9 | 1.5 | 1.6 | 0.3 | 6.1 |
| Pathum Thani | 25 | 209.5 | 410.9 | 671.5 | 104.8 | 3468.6 | 25 | 19.8 | 36.9 | 52.7 | 4.4 | 267.9 |
| Samut Prakan | 24 | 95.6 | 109.4 | 45.0 | 55.2 | 236.4 | 22 | 2.6 | 3.3 | 2.9 | 0.1 | 11.5 |
| Samut Sakhon | 19 | 132.1 | 149.0 | 65.4 | 70.7 | 350.5 | 18 | 2.0 | 2.2 | 1.2 | 0.6 | 4.5 |
| Nakhon Prathom | 17 | 126.1 | 130.5 | 40.5 | 66.7 | 209 | 17 | 1.3 | 1.4 | 0.8 | 0.1 | 2.8 |
| Total | 153 | 131.1 | 184.8 | 300 | 55.2 | 3468.6 | 143 | 2.7 | 9.8 | 25.5 | 0.0 | 267.9 |
