# Supplementary material for: Analysis of PM-bound polycyclic aromatic hydrocarbons exposure among motorcycle taxi drivers in six central provinces in Thailand in winter
Source: PLoS One. 2025 Dec 1;20(12):e0336587. doi: 10.1371/journal.pone.0336587 (PMC12668520; doi:10.1371/journal.pone.0336587)
Supplement: S7 Table — (DOCX) [file pone.0336587.s018.docx]

**S7 Table.** **Total BaP_eg_ for PM_2.5_-bound PAH in six provinces.**

| PAHs | TEF | BaP_eq_ for PM_2.5_-bound PAH (mg/m^3^) | | | | | |
| --- | --- | --- | --- | --- | --- | --- | --- |
|  |  | BKK | NBI | PTT | SPK | SKN | NPT |
| Napthalene | 0.001 | 1.1×10^-9^ | 5.5×10^-10^ | 7.7×10^-9^ | 1.5×10^-9^ | 1.1×10^-9^ | 8.5×10^-10^ |
| Acenapthene | 0.001 | 1.6×10^-9^ | 3.2×10^-11^ | 5.4×10^-9^ | 3.0×10^-11^ | 1.1×10^-10^ | UDL |
| Fluorene | 0.001 | 3.0×10^-10^ | 1.7×10^-10^ | 2.0×10^-12^ | 7.4×10^-11^ | 9.8×10^-11^ | 6.2×10^-11^ |
| Phenanthrene | 0.001 | 9.7×10^-10^ | 2.2×10^-10^ | 1.0×10^-10^ | 7.5×10^-10^ | 4.1×10^-10^ | 2.0×10^-10^ |
| Anthracene | 0.01 | 2.6×10^-9^ | UDL | 9.9×10^-9^ | UDL | UDL | UDL |
| Fluoranthene | 0.001 | 2.9×10^-10^ | 9.8×10^-11^ | 9.2×10^-9^ | 3.4×10^-10^ | 2.1×10^-10^ | 4.6×10^-11^ |
| Pyrene | 0.001 | 9.4×10^-10^ | 1.1×10^-10^ | 4.2×10^-9^ | 3.0×10^-10^ | 1.4×10^-10^ | 1.0×10^-10^ |
| Benzo(a)anthracene | 0.1 | 2.6×10^-8^ | 5.1×10^-9^ | 1.3×10^-7^ | 3.0×10^-10^ | UDL | UDL |
| Chrysene | 0.01 | 7.7×10^-9^ | 6.3×10^-10^ | 1.8×10^-8^ | 3.9×10^-10^ | 1.6×10^-10^ | UDL |
| Benzo(b)fluoranthene | 0.1 | 9.0×10^-9^ | 2.0×10^-9^ | 1.3×10^-7^ | 3.3×10^-9^ | UDL | UDL |
| Benzo(k)fluoranthene | 0.1 | 1.5×10^-8^ | 1.5×10^-8^ | 6.0×10^-8^ | 1.5×10^-8^ | 1.8×10^-8^ | 1.5×10^-8^ |
| Benzo(a)pyrene | 1 | 5.6×10^-8^ | 4.0×10^-9^ | 1.1×10^-6^ | 2.7×10^-8^ | UDL | UDL |
| Dibenzo(ah)anthracene | 1 | 1.0×10^-9^ | UDL | 4.1×10^-7^ | 5.0×10^-9^ | UDL | UDL |
| Benzo(ghi)perylene | 0.01 | 1.0×10^-11^ | UDL | 4.1×10^-9^ | 5.0×10^-11^ | UDL | UDL |
| Indeno(123-cd)pyrene | 0.1 | UDL | UDL | 8.3×10^-8^ | 6.6×10^-9^ | UDL | UDL |
| Total BaP_eq_ |  | 1.2×10^-7^ | 2.8×10^-8^ | 2.0×10^-6^ | 6.1×10^-8^ | 2.0×10^-8^ | 1.7×10^-8^ |

UDL= Under Detection Limit
