# Supplementary material for: Analysis of PM-bound polycyclic aromatic hydrocarbons exposure among motorcycle taxi drivers in six central provinces in Thailand in winter
Source: PLoS One. 2025 Dec 1;20(12):e0336587. doi: 10.1371/journal.pone.0336587 (PMC12668520; doi:10.1371/journal.pone.0336587)
Supplement: S8 Table — (DOCX) [file pone.0336587.s019.docx]

**S8 Table.** **Parameters used for calculating LADD and ILCR.**

| Parameters | Units | BKK | NBI | PTT | SPK | SKN | NPT | References |
| --- | --- | --- | --- | --- | --- | --- | --- | --- |
| Total BaP_eq_ PM_10_-bound PAH | mg/m^3^ | 4.1×10^-7^ | 2.6×10^-8^ | 1.2×10^-6^ | 6.7×10^-8^ | 2.5×10^-8^ | 1.7×10^-8^ | in this study |
| Total BaP_eq_ PM_2.5_-bound PAH | mg/m^3^ | 1.2×10^-7^ | 2.8×10^-8^ | 2.0×10^-6^ | 6.1×10^-8^ | 2.0×10^-8^ | 1.7×10^-8^ | in this study |
| ET | hours/day | 12.3 | 12.0 | 11.0 | 12.2 | 12.7 | 11.0 | in this study |
| EF | days/year | 316.8 | 321.6 | 316.8 | 307.2 | 316.8 | 312.0 | in this study |
| ED | years | 10.9 | 11.3 | 9.8 | 12.3 | 8.1 | 14.3 | in this study |
| BW | kg | 70.9 | 75.6 | 69.0 | 71.2 | 68.9 | 71.2 | in this study |
| IR | m^3^/day | 15.3 | 15.3 | 15.3 | 15.3 | 15.3 | 15.3 | [14] |
| AT | days | 25,500 | 25,500 | 25,500 | 25,500 | 25,500 | 25,500 | [15, 16] |
| CSF | mg/kg∙day | 3.14 | 3.14 | 3.14 | 3.14 | 3.14 | 3.14 | [15, 16] |

**References**

14. Agency for Toxic Substances and Disease Registry. Guidance for inhalation exposures. Atlanta: U.S. Department of Health and Human Services; 2020.

15. Li Z, Hao Q, Yue J, Qin J, Dong C, Li Y, et al. Pollution characteristics and health risk assessment of PM2.5-bound polycyclic aromatic hydrocarbons (PAHs), nitro-PAHs, and halogenated-PAHs in Shanxi, China. Air Quality, Atmosphere & Health. 2023.

16. Rajeev P, Singh AK, Singh GK, Vaishya RC, Gupta T. Chemical characterization, source identification and health risk assessment of polycyclic aromatic hydrocarbons in ambient particulate matter over central Indo-Gangetic Plain. Urban Climate. 2021;35:100755.
