# Supplementary material for: Analysis of PM-bound polycyclic aromatic hydrocarbons exposure among motorcycle taxi drivers in six central provinces in Thailand in winter
Source: PLoS One. 2025 Dec 1;20(12):e0336587. doi: 10.1371/journal.pone.0336587 (PMC12668520; doi:10.1371/journal.pone.0336587)
Supplement: S9 Table — (DOCX) [file pone.0336587.s020.docx]

**S9 Table.** **The concentration of PM_10_-bound PAHs (ng/m^3^).**

| Chemical | BKK | | | | | NBI | | | | |  | PTT | |  |  |  | SPK | |  |  |  | SKN | |  |  |  | NPT | |  |  |  | Total | |  |  |
| --- | --- | --- | --- | --- | --- | --- | --- | --- | --- | --- | --- | --- | --- | --- | --- | --- | --- | --- | --- | --- | --- | --- | --- | --- | --- | --- | --- | --- | --- | --- | --- | --- | --- | --- | --- |
|  | Median | Mean | SD | Min. | Max. | Median | Mean | SD | Min. | Max. | Median | Mean | SD | Min. | Max. | Median | Mean | SD | Min. | Max. | Median | Mean | SD | Min. | Max. | median | Mean | SD | Min. | Max. | Median | Mean | SD | Min. | Max. |
| Napthalene | 1.788 | 3.309 | 3.588 | 0.188 | 14.857 | 0.729 | 0.779 | 0.353 | 0.336 | 1.396 | 8.466 | 12.784 | 13.697 | 1.439 | 66.144 | 1.006 | 1.09 | 0.757 | 0.063 | 3.836 | 0.764 | 0.822 | 0.461 | 0 | 1.667 | 0.624 | 0.888 | 0.592 | 0.144 | 2.242 | 1.229 | 3.567 | 7.037 | 0 | 66.144 |
| Acenapthene | 0 | 0.805 | 1.592 | 0 | 6.804 | 0 | 0 | 0 | 0 | 0 | 0.428 | 2.578 | 3.51 | 0 | 11.573 | 0 | 0 | 0 | 0 | 0 | 0 | 0 | 0 | 0 | 0 | 0 | 0 | 0 | 0 | 0 | 0 | 0.671 | 1.868 | 0 | 11.573 |
| Fluorene | 0.061 | 0.325 | 0.507 | 0 | 2.265 | 0 | 0.174 | 0.338 | 0 | 1.068 | 0.401 | 1.31 | 1.995 | 0 | 6.679 | 0 | 0.202 | 0.448 | 0 | 1.424 | 0 | 0 | 0 | 0 | 0 | 0 | 0.018 | 0.056 | 0 | 0.216 | 0 | 0.371 | 0.952 | 0 | 6.679 |
| Phenanthrene | 0.668 | 1.379 | 2.348 | 0 | 13.848 | 0.227 | 0.317 | 0.235 | 0 | 0.713 | 2.14 | 2.973 | 3.525 | 0 | 14.068 | 0.731 | 0.783 | 0.567 | 0 | 1.856 | 0.213 | 0.361 | 0.351 | 0 | 1.245 | 0.252 | 0.421 | 0.543 | 0 | 2.242 | 0.549 | 1.184 | 2.133 | 0 | 14.068 |
| Anthracene | 0 | 0.012 | 0.051 | 0 | 0.298 | 0 | 0 | 0 | 0 | 0 | 4.955 | 7.804 | 9.713 | 0 | 38.283 | 0 | 0 | 0 | 0 | 0 | 0 | 0 | 0 | 0 | 0 | 0 | 0 | 0 | 0 | 0 | 0 | 1.222 | 4.714 | 0 | 38.283 |
| Fluoranthene | 0.776 | 1.114 | 1.52 | 0 | 7.167 | 0 | 0.044 | 0.071 | 0 | 0.223 | 0 | 1.004 | 1.559 | 0 | 4.654 | 0 | 0.211 | 0.386 | 0 | 1.525 | 0 | 0.093 | 0.217 | 0 | 0.692 | 0 | 0.015 | 0.062 | 0 | 0.248 | 0 | 0.582 | 1.182 | 0 | 7.167 |
| Pyrene | 1.099 | 1.772 | 2.618 | 0 | 12.683 | 0 | 0.096 | 0.138 | 0 | 0.391 | 3.562 | 3.606 | 3.397 | 0 | 13.934 | 0.13 | 0.324 | 0.458 | 0 | 1.724 | 0 | 0.108 | 0.24 | 0 | 0.764 | 0 | 0.113 | 0.2 | 0 | 0.744 | 0.282 | 1.246 | 2.364 | 0 | 13.934 |
| Benzo(a)anthracene | 0 | 0.423 | 1.599 | 0 | 9.963 | 0 | 0 | 0 | 0 | 0 | 0.881 | 1.419 | 1.805 | 0 | 8.523 | 0 | 0 | 0 | 0 | 0 | 0 | 0 | 0 | 0 | 0 | 0 | 0 | 0 | 0 | 0 | 0 | 0.363 | 1.254 | 0 | 9.963 |
| Chrysene | 0 | 0.998 | 3.751 | 0 | 23.422 | 0 | 0.072 | 0.298 | 0 | 1.228 | 1.448 | 1.587 | 1.184 | 0 | 5.043 | 0 | 0 | 0 | 0 | 0 | 0 | 0 | 0 | 0 | 0 | 0 | 0 | 0 | 0 | 0 | 0 | 0.589 | 2.286 | 0 | 23.422 |
| Benzo(b)fluoranthene | 0 | 0.344 | 0.906 | 0 | 4.916 | 0 | 0.013 | 0.054 | 0 | 0.223 | 1.021 | 1.263 | 0.997 | 0 | 4.251 | 0 | 0.05 | 0.252 | 0 | 1.26 | 0 | 0 | 0 | 0 | 0 | 0 | 0 | 0 | 0 | 0 | 0 | 0.322 | 0.785 | 0 | 4.916 |
| Benzo(k)fluoranthene | 0.185 | 0.243 | 0.225 | 0 | 1.398 | 0.168 | 0.157 | 0.029 | 0.116 | 0.223 | 0.264 | 0.419 | 0.497 | 0 | 2.043 | 0.134 | 0.174 | 0.138 | 0 | 0.796 | 0.198 | 0.185 | 0.041 | 0.133 | 0.277 | 0.147 | 0.158 | 0.034 | 0.138 | 0.264 | 0.169 | 0.232 | 0.255 | 0 | 2.043 |
| Benzo(a)pyrene | 0 | 0.284 | 0.963 | 0 | 5.943 | 0 | 0.007 | 0.027 | 0 | 0.112 | 0.458 | 0.581 | 0.492 | 0 | 1.889 | 0 | 0.042 | 0.212 | 0 | 1.061 | 0 | 0.005 | 0.018 | 0 | 0.069 | 0 | 0 | 0 | 0 | 0 | 0 | 0.194 | 0.625 | 0 | 5.943 |
| Dibenzo(ah)anthracene | 0 | 0.004 | 0.021 | 0 | 0.132 | 0 | 0 | 0 | 0 | 0 | 0 | 0.12 | 0.402 | 0 | 1.653 | 0 | 0 | 0 | 0 | 0 | 0 | 0 | 0 | 0 | 0 | 0 | 0 | 0 | 0 | 0 | 0 | 0.02 | 0.162 | 0 | 1.653 |
| Benzo(ghi)perylene | 0 | 0.004 | 0.021 | 0 | 0.132 | 0 | 0 | 0 | 0 | 0 | 0 | 0.12 | 0.402 | 0 | 1.653 | 0 | 0 | 0 | 0 | 0 | 0 | 0 | 0 | 0 | 0 | 0 | 0 | 0 | 0 | 0 | 0 | 0.02 | 0.162 | 0 | 1.653 |
| Indeno(123-cd)pyrene | 0 | 0.021 | 0.144 | 0 | 0.984 | 0 | 0 | 0 | 0 | 0 | 0 | 0.248 | 0.866 | 0 | 3.779 | 0 | 0 | 0 | 0 | 0 | 0 | 0 | 0 | 0 | 0 | 0 | 0 | 0 | 0 | 0 | 0 | 0.046 | 0.356 | 0 | 3.779 |
| Total PAHs | 6.432 | 11.051 | 14.449 | 0.478 | 78.832 | 1.508 | 1.66 | 1.016 | 0.514 | 4.019 | 28.328 | 38.435 | 29.202 | 6.006 | 111.068 | 2.674 | 2.877 | 2.067 | 0.063 | 9.35 | 1.324 | 1.576 | 1.104 | 0.14 | 3.96 | 1.216 | 1.614 | 1.214 | 0.288 | 5.012 | 3.441 | 10.73 | 18.899 | 0.063 | 111.068 |
