# Supplementary material for: Analysis of PM-bound polycyclic aromatic hydrocarbons exposure among motorcycle taxi drivers in six central provinces in Thailand in winter
Source: PLoS One. 2025 Dec 1;20(12):e0336587. doi: 10.1371/journal.pone.0336587 (PMC12668520; doi:10.1371/journal.pone.0336587)
Supplement: S10 Table — (DOCX) [file pone.0336587.s021.docx]

**S10 Table.** **The concentration of PM_2.5_-bound PAHs (ng/m^3^).**

| Chemical | BKK | | | | | NBI | | | | |  | PTT | |  |  |  | SPK | |  |  |  | SKN | |  |  |  | NPT | |  |  |  | Total | |  |  |
| --- | --- | --- | --- | --- | --- | --- | --- | --- | --- | --- | --- | --- | --- | --- | --- | --- | --- | --- | --- | --- | --- | --- | --- | --- | --- | --- | --- | --- | --- | --- | --- | --- | --- | --- | --- |
|  | Median | Mean | SD | Min. | Max. | Median | Mean | SD | Min. | Max. | Median | Mean | SD | Min. | Max. | Median | Mean | SD | Min. | Max. | Median | Mean | SD | Min. | Max. | median | Mean | SD | Min. | Max. | Median | Mean | SD | Min. | Max. |
| Napthalene | 0.667 | 1.096 | 1.192 | 0 | 5.061 | 14 | 0.552 | 0.427 | 0.109 | 1.371 | 25 | 7.661 | 5.054 | 0 | 22.73 | 0.936 | 1.484 | 2.048 | 0 | 10.182 | 0.784 | 1.055 | 0.87 | 0.22 | 3.415 | 0.944 | 0.848 | 0.421 | 0 | 1.426 | 0.944 | 2.216 | 3.451 | 0 | 22.73 |
| Acenapthene | 0 | 1.55 | 3.338 | 0 | 11.663 | 0.418 | 0.032 | 0.12 | 0 | 0.451 | 8.585 | 5.436 | 3.693 | 0.104 | 14.018 | 0 | 0.03 | 0.14 | 0 | 0.658 | 0 | 0.113 | 0.431 | 0 | 1.829 | 0 | 0 | 0 | 0 | 0 | 0 | 1.482 | 3.119 | 0 | 14.018 |
| Fluorene | 0.186 | 0.303 | 0.39 | 0 | 1.599 | 0 | 0.168 | 0.253 | 0 | 0.877 | 4.628 | 0.002 | 0.01 | 0 | 0.052 | 0 | 0.074 | 0.202 | 0 | 0.686 | 0 | 0.098 | 0.199 | 0 | 0.652 | 0 | 0.062 | 0.081 | 0 | 0.274 | 0 | 0.147 | 0.284 | 0 | 1.599 |
| Phenanthrene | 0.358 | 0.974 | 1.23 | 0 | 5.498 | 0.084 | 0.219 | 0.313 | 0 | 0.877 | 0 | 1.036 | 2.337 | 0 | 8.842 | 0.866 | 0.749 | 0.429 | 0 | 1.427 | 0.503 | 0.409 | 0.242 | 0 | 0.786 | 0.204 | 0.197 | 0.176 | 0 | 0.599 | 0.29 | 0.713 | 1.252 | 0 | 8.842 |
| Anthracene | 0 | 0.26 | 0.553 | 0 | 2.219 | 0.09 | 0 | 0 | 0 | 0 | 0 | 0.992 | 2.396 | 0 | 11.015 | 0 | 0 | 0 | 0 | 0 | 0 | 0 | 0 | 0 | 0 | 0 | 0 | 0 | 0 | 0 | 0 | 0.259 | 1.094 | 0 | 11.015 |
| Fluoranthene | 0 | 0.286 | 1.232 | 0 | 8.122 | 0 | 0.098 | 0.255 | 0 | 0.823 | 0 | 9.195 | 37.188 | 0 | 187.444 | 0 | 0.337 | 0.609 | 0 | 2.59 | 0 | 0.206 | 0.484 | 0 | 2 | 0 | 0.046 | 0.088 | 0 | 0.3 | 0 | 1.794 | 15.685 | 0 | 187.444 |
| Pyrene | 0 | 0.94 | 2.496 | 0 | 15.057 | 0 | 0.11 | 0.25 | 0 | 0.768 | 1.48 | 4.237 | 3.696 | 0 | 13.116 | 0.1 | 0.295 | 0.373 | 0 | 1.317 | 0 | 0.141 | 0.205 | 0 | 0.647 | 0 | 0.1 | 0.154 | 0 | 0.411 | 0 | 1.135 | 2.556 | 0 | 15.057 |
| Benzo(a)anthracene | 0 | 0.261 | 0.599 | 0 | 2.345 | 0 | 0.051 | 0.15 | 0 | 0.548 | 3.057 | 1.302 | 2.158 | 0 | 10.736 | 0 | 0.003 | 0.014 | 0 | 0.066 | 0 | 0 | 0 | 0 | 0 | 0 | 0 | 0 | 0 | 0 | 0 | 0.319 | 1.06 | 0 | 10.736 |
| Chrysene | 0 | 0.765 | 1.656 | 0 | 6.058 | 0 | 0.063 | 0.166 | 0 | 0.555 | 0.611 | 1.794 | 2.185 | 0 | 10.022 | 0 | 0.039 | 0.182 | 0 | 0.856 | 0 | 0.016 | 0.067 | 0 | 0.286 | 0 | 0 | 0 | 0 | 0 | 0 | 0.579 | 1.458 | 0 | 10.022 |
| Benzo(b)fluoranthene | 0 | 0.09 | 0.251 | 0 | 1.543 | 0 | 0.02 | 0.059 | 0 | 0.219 | 1.085 | 1.302 | 1.872 | 0 | 8.784 | 0 | 0.033 | 0.154 | 0 | 0.724 | 0 | 0 | 0 | 0 | 0 | 0 | 0 | 0 | 0 | 0 | 0 | 0.264 | 0.92 | 0 | 8.784 |
| Benzo(k)fluoranthene | 0.164 | 0.151 | 0.135 | 0 | 0.661 | 0 | 0.147 | 0.049 | 0.109 | 0.274 | 0.67 | 0.601 | 1.489 | 0 | 7.645 | 0.137 | 0.154 | 0.122 | 0 | 0.592 | 0.188 | 0.175 | 0.062 | 0 | 0.289 | 0.145 | 0.154 | 0.025 | 0.136 | 0.218 | 0.149 | 0.233 | 0.642 | 0 | 7.645 |
| Benzo(a)pyrene | 0 | 0.056 | 0.212 | 0 | 1.377 | 0.12 | 0.004 | 0.015 | 0 | 0.055 | 0.234 | 1.141 | 1.806 | 0 | 9.001 | 0 | 0.027 | 0.126 | 0 | 0.592 | 0 | 0 | 0 | 0 | 0 | 0 | 0 | 0 | 0 | 0 | 0 | 0.222 | 0.865 | 0 | 9.001 |
| Dibenzo(ah)anthracene | 0 | 0.001 | 0.008 | 0 | 0.055 | 0 | 0 | 0 | 0 | 0 | 0.493 | 0.414 | 1.983 | 0 | 9.922 | 0 | 0.005 | 0.026 | 0 | 0.121 | 0 | 0 | 0 | 0 | 0 | 0 | 0 | 0 | 0 | 0 | 0 | 0.074 | 0.83 | 0 | 9.922 |
| Benzo(ghi)perylene | 0 | 0.001 | 0.008 | 0 | 0.055 | 0 | 0 | 0 | 0 | 0 | 0 | 0.414 | 1.983 | 0 | 9.922 | 0 | 0.005 | 0.026 | 0 | 0.121 | 0 | 0 | 0 | 0 | 0 | 0 | 0 | 0 | 0 | 0 | 0 | 0.074 | 0.83 | 0 | 9.922 |
| Indeno(123-cd)pyrene | 0 | 0 | 0 | 0 | 0 | 0 | 0 | 0 | 0 | 0 | 0 | 0.825 | 2.397 | 0 | 11.17 | 0 | 0.066 | 0.309 | 0 | 1.448 | 0 | 0 | 0 | 0 | 0 | 0 | 0 | 0 | 0 | 0 | 0 | 0.154 | 1.04 | 0 | 11.17 |
| Total PAHs | 4.555 | 6.733 | 7.1 | 0 | 34.488 | 0 | 1.464 | 1.601 | 0.327 | 6.142 | 0 | 36.869 | 52.674 | 4.421 | 267.922 | 2.619 | 3.296 | 2.897 | 0.137 | 11.503 | 2.042 | 2.212 | 1.182 | 0.584 | 4.497 | 1.307 | 1.406 | 0.764 | 0.149 | 2.846 | 2.673 | 9.755 | 25.46 | 0 | 267.922 |
