# Supplementary material for: Analysis of PM-bound polycyclic aromatic hydrocarbons exposure among motorcycle taxi drivers in six central provinces in Thailand in winter
Source: PLoS One. 2025 Dec 1;20(12):e0336587. doi: 10.1371/journal.pone.0336587 (PMC12668520; doi:10.1371/journal.pone.0336587)
Supplement: S11 Table — (DOCX) [file pone.0336587.s022.docx]

**S11 Table.** **Association between categorical variables and FVC (%predicted).**

| Parameter | Independent Variables | Test | P-Value |
| --- | --- | --- | --- |
| FVC (%predicted) | Province | ANOVA | <0.001** |
| FVC (%predicted) | Workstation | ANOVA | <0.001** |
| FVC (%predicted) | Marital status | ANOVA | 0.990 |
| FVC (%predicted) | Helmet type | ANOVA | 0.237 |
| FVC (%predicted) | Hairy pet | ANOVA | 0.041* |
| FVC (%predicted) | Smoke | ANOVA | 0.112 |
| FVC (%predicted) | Mask type | ANOVA | 0.246 |
| FVC (%predicted) | Education | ANOVA | 0.295 |
| FVC (%predicted) | Age group | ANOVA | <0.001** |
| FVC (%predicted) | BMI | ANOVA | 0.793 |
| FVC (%predicted) | Work experience | ANOVA | 0.151 |
| FVC (%predicted) | Frequency of physical activity | ANOVA | 0.901 |
| FVC (%predicted) | Place of physical activity | ANOVA | 0.842 |
| FVC (%predicted) | Gender | t-test | 0.122 |
| FVC (%predicted) | Secondhand smoker | t-test | 0.120 |
| FVC (%predicted) | History of the covid-19 | t-test | 0.422 |
| FVC (%predicted) | Vaccine covid-19 | t-test | 0.284 |
| FVC (%predicted) | Diabetes | t-test | 0.052 |
| FVC (%predicted) | Hypertension | t-test | <0.001** |
| FVC (%predicted) | Nasal allergy | t-test | 0.109 |
| FVC (%predicted) | Allergy skin rash | t-test | 0.221 |
| FVC (%predicted) | Chest pain | t-test | 0.010** |
| FVC (%predicted) | History of asthma | t-test | 0.135 |
| FVC (%predicted) | History of tuberculosis | t-test | 0.183 |
| FVC (%predicted) | History of allergy | t-test | 0.004** |
| FVC (%predicted) | Neuromuscular | t-test | 0.054 |
| FVC (%predicted) | Garbage disposal | t-test | 0.562 |
| FVC (%predicted) | Road type | t-test | 0.012* |
| FVC (%predicted) | Mosquito repellent coil | t-test | 0.182 |
| FVC (%predicted) | Insect repellent spray | t-test | 0.379 |
| FVC (%predicted) | Incense smoke in the house | t-test | 0.137 |
| FVC (%predicted) | Cooking with firewood | t-test | 0.414 |
| FVC (%predicted) | Driving type | t-test | 0.425 |
| FVC (%predicted) | Break period | t-test | 0.059 |
| FVC (%predicted) | Persistent cough | t-test | 0.967 |
| FVC (%predicted) | Persistent phlegm | t-test | 0.084 |
| FVC (%predicted) | Chronic bronchitis | t-test | 0.223 |
| FVC (%predicted) | Acute bronchitis | t-test | 0.525 |
| FVC (%predicted) | Bronchial asthma | t-test | 0.132 |
| FVC (%predicted) | Chronic Obstructive Pulmonary Disease | t-test | 0.266 |

* p-value < 0.05, **p-value<0.01
