# Supplementary material for: Analysis of PM-bound polycyclic aromatic hydrocarbons exposure among motorcycle taxi drivers in six central provinces in Thailand in winter
Source: PLoS One. 2025 Dec 1;20(12):e0336587. doi: 10.1371/journal.pone.0336587 (PMC12668520; doi:10.1371/journal.pone.0336587)
Supplement: S12 Table — (DOCX) [file pone.0336587.s023.docx]

**S12 Table.** **Association between categorical variables and FEV1 (%predicted).**

| Parameter | Independent Variables | Test | P-Value |
| --- | --- | --- | --- |
| FEV1 (%predicted) | Province | ANOVA | 0.576 |
| FEV1 (%predicted) | Workstation | ANOVA | 0.255 |
| FEV1 (%predicted) | Marital status | ANOVA | 0.450 |
| FEV1 (%predicted) | Helmet type | ANOVA | 0.202 |
| FEV1 (%predicted) | Hairy pet | ANOVA | 0.065 |
| FEV1 (%predicted) | Smoke | ANOVA | 0.389 |
| FEV1 (%predicted) | Mask type | ANOVA | 0.759 |
| FEV1 (%predicted) | Education | ANOVA | 0.147 |
| FEV1 (%predicted) | Age group | ANOVA | <0.001** |
| FEV1 (%predicted) | BMI | ANOVA | 0.686 |
| FEV1 (%predicted) | Work experience | ANOVA | 0.099 |
| FEV1 (%predicted) | Frequency of physical activity | ANOVA | 0.584 |
| FEV1 (%predicted) | Place of physical activity | ANOVA | 0.671 |
| FEV1 (%predicted) | Gender | t-test | 0.421 |
| FEV1 (%predicted) | Secondhand smoker | t-test | 0.366 |
| FEV1 (%predicted) | History of the covid-19 | t-test | 0.647 |
| FEV1 (%predicted) | Vaccine covid-19 | t-test | 0.739 |
| FEV1 (%predicted) | Diabetes | t-test | 0.312 |
| FEV1 (%predicted) | Hypertension | t-test | 0.029* |
| FEV1 (%predicted) | Nasal allergy | t-test | 0.455 |
| FEV1 (%predicted) | Allergy skin rash | t-test | 0.595 |
| FEV1 (%predicted) | Chest pain | t-test | 0.224 |
| FEV1 (%predicted) | History of asthma | t-test | 0.018* |
| FEV1 (%predicted) | History of tuberculosis | t-test | 0.209 |
| FEV1 (%predicted) | History of allergy | t-test | 0.012* |
| FEV1 (%predicted) | Neuromuscular | t-test | 0.013* |
| FEV1 (%predicted) | Garbage disposal | t-test | 0.109 |
| FEV1 (%predicted) | Road type | t-test | 0.832 |
| FEV1 (%predicted) | Mosquito repellent coil | t-test | 0.240 |
| FEV1 (%predicted) | Insect repellent spray | t-test | 0.883 |
| FEV1 (%predicted) | Incense smoke in the house | t-test | 0.812 |
| FEV1 (%predicted) | Cooking with firewood | t-test | 0.594 |
| FEV1 (%predicted) | Driving type | t-test | 0.335 |
| FEV1 (%predicted) | Break period | t-test | 0.242 |
| FEV1 (%predicted) | Persistent cough | t-test | 0.882 |
| FEV1 (%predicted) | Persistent phlegm | t-test | 0.182 |
| FEV1 (%predicted) | Chronic bronchitis | t-test | 0.048* |
| FEV1 (%predicted) | Acute bronchitis | t-test | 0.122 |
| FEV1 (%predicted) | Bronchial asthma | t-test | 0.130 |
| FEV1 (%predicted) | Chronic Obstructive Pulmonary Disease | t-test | 0.258 |

* p-value < 0.05, **p-value<0.01
