# Supplementary material for: Analysis of PM-bound polycyclic aromatic hydrocarbons exposure among motorcycle taxi drivers in six central provinces in Thailand in winter
Source: PLoS One. 2025 Dec 1;20(12):e0336587. doi: 10.1371/journal.pone.0336587 (PMC12668520; doi:10.1371/journal.pone.0336587)
Supplement: S13 Table — (DOCX) [file pone.0336587.s024.docx]

**S13 Table.** **Association between categorical variables and FEV1/FVC (%predicted).**

| Parameter | Independent Variables | Test | P-Value |
| --- | --- | --- | --- |
| FEV1/FVC (%predicted) | Province | ANOVA | <0.001** |
| FEV1/FVC (%predicted) | Workstation | ANOVA | <0.001** |
| FEV1/FVC (%predicted) | Marital status | ANOVA | 0.834 |
| FEV1/FVC (%predicted) | Helmet type | ANOVA | 0.644 |
| FEV1/FVC (%predicted) | Hairy pet | ANOVA | 0.737 |
| FEV1/FVC (%predicted) | Smoke | ANOVA | 0.115 |
| FEV1/FVC (%predicted) | Mask type | ANOVA | 0.163 |
| FEV1/FVC (%predicted) | Education | ANOVA | 0.387 |
| FEV1/FVC (%predicted) | Age group | ANOVA | 0.945 |
| FEV1/FVC (%predicted) | BMI | ANOVA | 0.962 |
| FEV1/FVC (%predicted) | Work experience | ANOVA | 0.982 |
| FEV1/FVC (%predicted) | Frequency of physical activity | ANOVA | 0.375 |
| FEV1/FVC (%predicted) | Place of physical activity | ANOVA | 0.554 |
| FEV1/FVC (%predicted) | Gender | t-test | 0.330 |
| FEV1/FVC (%predicted) | Secondhand smoker | t-test | 0.392 |
| FEV1/FVC (%predicted) | History of the covid-19 | t-test | 0.597 |
| FEV1/FVC (%predicted) | Vaccine covid-19 | t-test | 0.010* |
| FEV1/FVC (%predicted) | Diabetes | t-test | 0.299 |
| FEV1/FVC (%predicted) | Hypertension | t-test | 0.103 |
| FEV1/FVC (%predicted) | Nasal allergy | t-test | 0.197 |
| FEV1/FVC (%predicted) | Allergy skin rash | t-test | 0.275 |
| FEV1/FVC (%predicted) | Chest pain | t-test | 0.173 |
| FEV1/FVC (%predicted) | History of asthma | t-test | 0.013* |
| FEV1/FVC (%predicted) | History of tuberculosis | t-test | 0.326 |
| FEV1/FVC (%predicted) | History of allergy | t-test | 0.083 |
| FEV1/FVC (%predicted) | Neuromuscular | t-test | 0.917 |
| FEV1/FVC (%predicted) | Garbage disposal | t-test | 0.230 |
| FEV1/FVC (%predicted) | Road type | t-test | 0.002** |
| FEV1/FVC (%predicted) | Mosquito repellent coil | t-test | 0.725 |
| FEV1/FVC (%predicted) | Insect repellent spray | t-test | 0.272 |
| FEV1/FVC (%predicted) | Incense smoke in the house | t-test | 0.024* |
| FEV1/FVC (%predicted) | Cooking with firewood | t-test | 0.650 |
| FEV1/FVC (%predicted) | Driving type | t-test | 0.960 |
| FEV1/FVC (%predicted) | Break period | t-test | 0.347 |
| FEV1/FVC (%predicted) | Persistent cough | t-test | 0.454 |
| FEV1/FVC (%predicted) | Persistent phlegm | t-test | 0.039* |
| FEV1/FVC (%predicted) | Chronic bronchitis | t-test | 0.070 |
| FEV1/FVC (%predicted) | Acute bronchitis | t-test | 0.006** |
| FEV1/FVC (%predicted) | Bronchial asthma | t-test | 0.090 |
| FEV1/FVC (%predicted) | Chronic Obstructive Pulmonary Disease | t-test | 0.172 |

* p-value < 0.05, **p-value<0.01
