# Supplementary material for: Analysis of PM-bound polycyclic aromatic hydrocarbons exposure among motorcycle taxi drivers in six central provinces in Thailand in winter
Source: PLoS One. 2025 Dec 1;20(12):e0336587. doi: 10.1371/journal.pone.0336587 (PMC12668520; doi:10.1371/journal.pone.0336587)
Supplement: S14 Table — (DOCX) [file pone.0336587.s025.docx]

**S14 Table.** **Association between numerical variables and FVC (%predicted).**

| Parameter | Independent Variables | Pearson’s  Correlation (95% CI) | n | P-Value |
| --- | --- | --- | --- | --- |
| FVC (%predicted) | Cigarette (amount/day) | 0.049 (-0.045, 0.141) | 441 | 0.308 |
| FVC (%predicted) | Age (year) | -0.156 (-0.246, -0.063) | 441 | 0.001** |
| FVC (%predicted) | Income (baht/month) | 0.007 (-0.086, 0.100) | 441 | 0.884 |
| FVC (%predicted) | Age of start smoking (year) | 0.010 (-0.131, 0.150) | 195 | 0.894 |
| FVC (%predicted) | Duration of smoking (year) | -0.015 (-0.108, 0.079) | 441 | 0.755 |
| FVC (%predicted) | Waist circumference (cm) | -0.085 (-0.177, 0.009) | 441 | 0.075 |
| FVC (%predicted) | Height (m) | -0.039 (-0.132, 0.055) | 441 | 0.413 |
| FVC (%predicted) | Weight (kg) | -0.045 (-0.138, 0.049) | 441 | 0.346 |
| FVC (%predicted) | Body mass index (kg/m2) | -0.0299 (-0.123, 0.064) | 441 | 0.532 |
| FVC (%predicted) | Systolic blood pressure (mmHg) | -0.087 (-0.179, 0.006) | 441 | 0.067 |
| FVC (%predicted) | Diastolic blood pressure (mmHg) | -0.131 (-0.222, -0.038) | 441 | 0.006** |
| FVC (%predicted) | Work experience (year) | -0.096 (-0.187, -0.002) | 441 | 0.045* |
| FVC (%predicted) | Working time (hour/day) | -0.031 (-0.124, 0.062) | 441 | 0.511 |
| FVC (%predicted) | Working day (day/week) | -0.006 (-0.099, 0.087) | 441 | 0.900 |
| FVC (%predicted) | Outdoor time (hour/day) | 0.046 (-0.048, 0.138) | 441 | 0.340 |
| FVC (%predicted) | Break period (hour/day) | -0.081 (-0.173, 0.012) | 441 | 0.088 |
| FVC (%predicted) | Sleep time (hour/day) | -0.081 (-0.173, 0.013) | 441 | 0.091 |

* p-value < 0.05, **p-value<0.01
