# Supplementary material for: Analysis of PM-bound polycyclic aromatic hydrocarbons exposure among motorcycle taxi drivers in six central provinces in Thailand in winter
Source: PLoS One. 2025 Dec 1;20(12):e0336587. doi: 10.1371/journal.pone.0336587 (PMC12668520; doi:10.1371/journal.pone.0336587)
Supplement: S15 Table — (DOCX) [file pone.0336587.s026.docx]

**S15 Table.** **Association between numerical variables and FEV1 (%predicted).**

| Parameter | Independent Variables | Pearson’s Correlation  (95% CI) | n | P-Value |
| --- | --- | --- | --- | --- |
| FEV1 (%predicted) | Cigarette (amount/day) | -0.007 (-0.100, 0.087) | 441 | 0.888 |
| FEV1 (%predicted) | Age (year) | -0.146 (-0.236, -0.053) | 441 | 0.002** |
| FEV1 (%predicted) | Income (baht/month) | 0.044 (-0.050, 0.137) | 441 | 0.359 |
| FEV1 (%predicted) | Age of start smoking (year) | 0.008 (-0.133, 0.148) | 195 | 0.914 |
| FEV1 (%predicted) | Duration of smoking (year) | -0.083 (-0.175, 0.011) | 441 | 0.082 |
| FEV1 (%predicted) | Waist circumference (cm) | -0.078 (-0.170, 0.015) | 441 | 0.101 |
| FEV1 (%predicted) | Height (m) | -0.020 (-0.113, 0.073) | 441 | 0.674 |
| FEV1 (%predicted) | Weight (kg) | -0.035 (-0.128, 0.059) | 441 | 0.465 |
| FEV1 (%predicted) | Body mass index (kg/m2) | -0.024 (-0.117, 0.069) | 441 | 0.611 |
| FEV1 (%predicted) | Systolic blood pressure (mmHg) | -0.069 (-0.161, 0.025) | 441 | 0.148 |
| FEV1 (%predicted) | Diastolic blood pressure (mmHg) | -0.124 (-0.215, -0.031) | 441 | 0.009** |
| FEV1 (%predicted) | Work experience (year) | -0.074 (-0.166, 0.020) | 441 | 0.123 |
| FEV1 (%predicted) | Working time (hour/day) | -0.025 (-0.118, 0.069) | 441 | 0.603 |
| FEV1 (%predicted) | Working day (day/week) | -0.000 (-0.094, 0.093) | 441 | 0.995 |
| FEV1 (%predicted) | Outdoor time (hour/day) | 0.058 (-0.035, 0.151) | 441 | 0.220 |
| FEV1 (%predicted) | Break period (hour/day) | -0.047 (-0.140, 0.046) | 441 | 0.323 |
| FEV1 (%predicted) | Sleep time (hour/day) | -0.066 (-0.158, 0.027) | 441 | 0.166 |

* p-value < 0.05, **p-value<0.01
