# Supplementary material for: Analysis of PM-bound polycyclic aromatic hydrocarbons exposure among motorcycle taxi drivers in six central provinces in Thailand in winter
Source: PLoS One. 2025 Dec 1;20(12):e0336587. doi: 10.1371/journal.pone.0336587 (PMC12668520; doi:10.1371/journal.pone.0336587)
Supplement: S16 Table — (DOCX) [file pone.0336587.s027.docx]

**S16 Table.** **Association between numerical variables and FEV1/FVC (%predicted).**

| Parameter | Independent Variables | Pearson’s Correlation (95% CI) | n | P-Value |
| --- | --- | --- | --- | --- |
| FEV1/FVC (%predicted) | Cigarette (amount/day) | -0.106 (-0.198, -0.013) | 441 | 0.026* |
| FEV1/FVC (%predicted) | Age (year) | 0.022 (-0.072, 0.115) | 441 | 0.646 |
| FEV1/FVC (%predicted) | Income (baht/month) | 0.059 (-0.034, 0.152) | 441 | 0.215 |
| FEV1/FVC (%predicted) | Age of start smoking (year) | 0.001 (-0.140, 0.141) | 195 | 0.990 |
| FEV1/FVC (%predicted) | Duration of smoking (year) | -0.112 (-0.203, -0.019) | 441 | 0.018* |
| FEV1/FVC (%predicted) | Waist circumference (cm) | 0.001 (-0.092, 0.094) | 441 | 0.982 |
| FEV1/FVC (%predicted) | Height (m) | -0.015 (-0.108, 0.078) | 441 | 0.753 |
| FEV1/FVC (%predicted) | Weight (kg) | 0.003 (-0.091, 0.096) | 441 | 0.954 |
| FEV1/FVC (%predicted) | Body mass index (kg/m^2^) | 0.012 (-0.081, 0.106) | 441 | 0.794 |
| FEV1/FVC (%predicted) | Systolic blood pressure (mmHg) | 0.0434 (-0.050, 0.136) | 441 | 0.364 |
| FEV1/FVC (%predicted) | Diastolic blood pressure (mmHg) | 0.022 (-0.072, 0.115) | 441 | 0.651 |
| FEV1/FVC (%predicted) | Work experience (year) | 0.052 (-0.042, 0.145) | 441 | 0.276 |
| FEV1/FVC (%predicted) | Working time (hour/day) | 0.011 (-0.082, 0.104) | 441 | 0.815 |
| FEV1/FVC (%predicted) | Working day (day/week) | -0.011 (-0.104, 0.083) | 441 | 0.819 |
| FEV1/FVC (%predicted) | Outdoor time (hour/day) | 0.059 (-0.035, 0.151) | 441 | 0.220 |
| FEV1/FVC (%predicted) | Break period (hour/day) | 0.051 (-0.042, 0.144) | 441 | 0.283 |
| FEV1/FVC (%predicted) | Sleep time (hour/day) | -0.066 (-0.158, 0.027) | 441 | 0.166 |

* p-value < 0.05, **p-value<0.01
